# Complete sequences of organelle genomes from the medicinal plant *Rhazya stricta* (Apocynaceae) and contrasting patterns of mitochondrial genome evolution across asterids

**DOI:** 10.1186/1471-2164-15-405

**Published:** 2014-05-28

**Authors:** Seongjun Park, Tracey A Ruhlman, Jamal SM Sabir, Mohammed HZ Mutwakil, Mohammed N Baeshen, Meshaal J Sabir, Nabih A Baeshen, Robert K Jansen

**Affiliations:** Department of Integrative Biology, University of Texas at Austin, Austin, TX 78712 USA; Department of Biological Science, Faculty of Science, King Abdulaziz University, Jeddah, 21589 Saudi Arabia

**Keywords:** Asterids, Gene transfers, Medicinal plant, Organelle genomes, Harmal, Adfir

## Abstract

**Background:**

*Rhazya stricta* is native to arid regions in South Asia and the Middle East and is used extensively in folk medicine to treat a wide range of diseases. In addition to generating genomic resources for this medicinally important plant, analyses of the complete plastid and mitochondrial genomes and a nuclear transcriptome from *Rhazya* provide insights into inter-compartmental transfers between genomes and the patterns of evolution among eight asterid mitochondrial genomes.

**Results:**

The 154,841 bp plastid genome is highly conserved with gene content and order identical to the ancestral organization of angiosperms. The 548,608 bp mitochondrial genome exhibits a number of phenomena including the presence of recombinogenic repeats that generate a multipartite organization, transferred DNA from the plastid and nuclear genomes, and bidirectional DNA transfers between the mitochondrion and the nucleus. The mitochondrial genes *sdh3* and *rps14* have been transferred to the nucleus and have acquired targeting presequences. In the case of *rps14*, two copies are present in the nucleus; only one has a mitochondrial targeting presequence and may be functional. Phylogenetic analyses of both nuclear and mitochondrial copies of *rps14* across angiosperms suggests *Rhazya* has experienced a single transfer of this gene to the nucleus, followed by a duplication event. Furthermore, the phylogenetic distribution of gene losses and the high level of sequence divergence in targeting presequences suggest multiple, independent transfers of both *sdh3* and *rps14* across asterids. Comparative analyses of mitochondrial genomes of eight sequenced asterids indicates a complicated evolutionary history in this large angiosperm clade with considerable diversity in genome organization and size, repeat, gene and intron content, and amount of foreign DNA from the plastid and nuclear genomes.

**Conclusions:**

Organelle genomes of *Rhazya stricta* provide valuable information for improving the understanding of mitochondrial genome evolution among angiosperms. The genomic data have enabled a rigorous examination of the gene transfer events. *Rhazya* is unique among the eight sequenced asterids in the types of events that have shaped the evolution of its mitochondrial genome. Furthermore, the organelle genomes of *R. stricta* provide valuable genomic resources for utilizing this important medicinal plant in biotechnology applications.

**Electronic supplementary material:**

The online version of this article (doi: 10.1186/1471-2164-15-405) contains supplementary material, which is available to authorized users.

## Background

*Rhazya stricta* (Apocynaceae) is among the most economically important medicinal plants adapted to the arid regions of South Asia and the Middle East. Leaf extracts are prescribed in folk medicine for the treatment of various disorders including diabetes, sore throat, syphilis, helminthiasis, inflammatory conditions and rheumatism [1–3]. At least 100 alkaloids have been isolated and identified from this species [4, 5]. A recent study by Baeshen et al. [6] found that *R. stricta* extracts promoted apoptosis induction in breast cancer cells, suggesting its potential as a chemo-preventive or therapeutic agent. However, currently there are no genomic resources for *R. stricta* to facilitate the development of this species for therapeutic applications using a natural products genomics approach [7, 8].

The availability of plant genomic data has improved dramatically in recent years through the development of next-generation sequencing (NGS) technologies [9–11] and improved assembly methods [12]. Genomic resources that provide information about gene content and the metabolic pathways that produce compounds with pharmaceutical value are paramount to the potential for improvement and application of natural products as therapeutics.

Most plastid genomes have a quadripartite structure with an inverted repeat separated by large and small single-copy regions, an arrangement that is highly conserved across land plants [13]. Plastid genome sizes of photosynthetic land plants range from 107 to 217 kb and contain 101-118 different genes with majority of these coding for proteins involved in photosynthesis and gene expression along with transfer RNA (tRNA) and ribosomal RNA (rRNA) genes [14].

In contrast mitochondrial genomes are remarkably variable among land plants. These genomes are usually larger than plastid genomes, ranging from 105 kb in the moss *Physcomitrella patens*[15] to 11.3 Mb in the angiosperm *Silene conica*[16]. Despite their relatively large size, mitochondrial genomes contain fewer genes than their plastid counterparts; 37-83 different genes including protein coding, tRNA and rRNA genes [17]. Plant mitochondrial genomes map as circular or noncircular structures and have a dynamic, multipartite organization due to active recombination associated with repeat regions that can be as large as 109 kb in *Tripsacum dactyloides*[18–22]. Rearrangements in plant mitochondrial genomes are facilitated by homologs of bacterial DNA repair proteins such as *RecA* and *MutS*[23] and often result in chimeric open reading frames (ORFs) [22, 24]. One of the most prominent characteristics of plant mitochondrial genomes is the presence of foreign DNA including DNA that has been transferred from the plastid, nucleus and even genetic material from other species [25–29]. RNA editing is common in plant mitochondrial genomes and usually involves the conversion of cytidine (C) to uracil (U) [30, 31].

Among angiosperms there is a high frequency of organellar DNA transferred to the nucleus. Although most functional gene transfers from organelles to the nucleus occurred shortly after their endosymbiotic origin, gene transfer is an ongoing process [25]. While transfer of organellar DNA to the nucleus is rampant, acquisition of function is much less common. Transferred organelle genes must acquire nuclear expression elements and target peptides to shuttle the gene product back to the organelle (referred to as a transit peptide in the case of plastids or a presequence for mitochondria) [32]. Transferred organelle genes may acquire a novel targeting sequence or adopt one from an existing nuclear gene [33–35]. In a few cases, mitochondrial genes have been transferred without the acquisition of N-terminal sequences. In these cases, gene products may be targeted to the mitochondrion by internal features of the polypeptide [36].

This report provides the complete sequences of the plastid and mitochondrial genomes of *R. stricta*, a medicinally important species. Genome organization is characterized including identification of the gene transfers between the mitochondria and the nucleus. In addition, the mitochondrial genome organization of *R. stricta* is compared to seven previously published asterid genomes to examine patterns of organelle genome evolution across this large angiosperm clade.

## Results

### Plastid genome organization of Rhazya stricta

**Figure 1:**
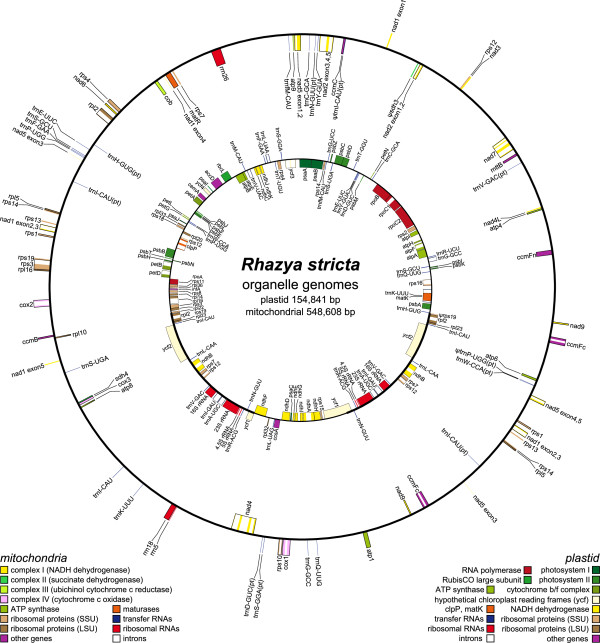
**Maps of the organelle genomes of** ***Rhazya stricta***. The inner and outer circles represent the plastid and mitochondrial genomes, respectively. Genes on the inside and outside of each map are transcribed clockwise and counterclockwise direction, respectively. The thick lines on the plastid map indicate the inverted repeats (IRa and IRb), which separate the genome into large and small singles copy region. Ψ denotes a pseudogene.

The *R. stricta* plastid genome is 154,841 bp in length with a pair of inverted repeats (IRa and IRb) of 25,513 bp separated by small and large single-copy (SSC and LSC) regions of 17,745 and 86,070 bp, respectively (Figure 1 and Table 1). The GC content is 37.6%, and the genome consists of 50.7% protein-coding genes, 41.6% non-coding regions, 1.8% tRNA and 5.8% rRNA genes. The *Rhazya* plastid genome encodes 114 genes, 16 of which are duplicated in the IR, for a total of 130 genes. There are 80 protein-coding genes, 30 tRNA and 4 rRNA genes (Table 1). The IR has expanded slightly at both the IR/LSC and IR/SSC boundaries relative to *N. tabacum*. The expansion encompassed the first 91 nucleotides of *rps19* in IRb, generating an *rps19* fragment in IRa, and extended into the SSC to include the stop codon of the *ndhF* gene. Mitochondrial-like gene sequences were not detected in the *Rhazya* plastid genome.

**Table 1:** **Characteristics of** ***Rhazya stricta*** **organelle genomes**

Genome characteristic	Plastid genome	Mitochondrial genome
Genome size (bp)	154,841	548,608
GC content (%)	37.6	43.7
Genes		
Protein coding genes	80 (5)	38 (6)
(%)	50.7	6.7
tRNA genes	30 (7)	12
(%)	1.8	0.2
rRNA genes	4 (4)	3
(%)	5.8	1.0
Introns		
*cis*-spliced group	20 (5)	18
*trans*-spliced group	1	6
Plastid-derived		
Protein-coding genes		4 (2)
tRNA genes		7 (1)

The numbers in parentheses indicate the number of genes duplicated in repeat region.

### Mitochondrial genome organization of Rhazya stricta

#### Genome size and content

The *R. stricta* mitochondrial genome assembled into a single master chromosome of 548,608 bp (Figure 1). The GC content is 43.7%, and the genome consists of 6.7% protein-coding genes, 92.1% intergenic spacers, 0.2% tRNA and 1.0% rRNA genes (Table 1). The genome contains 53 genes including 38 protein-coding genes, 12 tRNAs, and 3 rRNAs (Table 1; Additional file 1: Table S1). Two identical copies of the genes *nad9*, *ccmFc*, *rpl15*, *rps1*, *rps13* and *rps14* were identified in two large repeats (Additional file 1: Table S1). The ribosomal protein genes *rps2* and *rps11* are absent and respiratory protein gene *sdh3* appeared to be a pseudogene as it lacks a start codon. The *rps14* gene has an 8 bp deletion near the 3’ end resulting in a frame shift that truncates the sequence relative to other angiosperms. The N-terminal portion of the *atp6* gene is truncated, having lost 125 amino acids relative to the *atp6* gene of *Nicotiana tabacum*. The *Rhazya atp6* has only a small portion of the gene containing the conserved ATP synthase F0 subunit 6 domain (pfam00119). The *cox1* group I intron was identified as well as 23 group II introns, six of which require *trans*-splicing (Table 1).

#### Repeat structure

BlastN analysis of the *Rhazya* mitochondrial genome against itself revealed 77,887 bp of repetitive DNA, ranging from 39 to 36,624 bp in length (Additional file 1: Table S2). The repetitive DNA constitutes 14.2% of the genome including 12.9% large (>1 kb), 0.6% intermediate (100-1000 bp) and 0.7% small (<100 bp) repeats. Mapping of corrected PacBio reads against the mitochondrial genome revealed conflicts between the PacBio reads and the assembled genome suggesting the existence of subgenomic circles (Additional file 2: Figure S1A); the long PacBio reads spanned the junctions of the repeat regions in the assembled master chromosome. The conflicts were associated with two large repeats (36,251 and 32,072 bp) and five intermediate-sized repeats (559, 508, 281, 252, and 124 bp) (Additional file 2: Figure S1B). Most of the repeats clustered together sequentially in twos, threes or fives. PCR confirmed the existence of isomeric and/or subgenomic circles that arose as a result of repeat-mediated homologous recombination among the seven repeat families (Additional file 2: Figure S2).

#### Transposable elements

**Figure 2:**
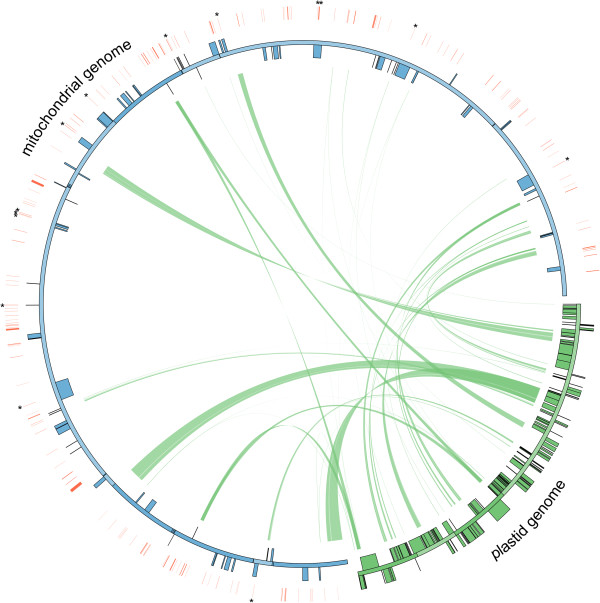
**Schematic representation of transfers of plastid DNA and transposable elements into the mitochondrial genome of** ***Rhazya stricta***. Each green line within the circle shows the regions of the plastid genome that have been inserted into different locations of the mitochondrial genome. Red lines outside of the mitochondrial genome indicate the location of integrated transposable elements (TEs) and asterisks indicate TEs that have inserted into genic regions. Genes indicated as blue and green boxes on the inside and outside of maps are transcribed clockwise and counterclockwise direction, respectively.

The *Rhazya* mitochondrial genome contains 16,008 bp (2.9%) of transposable elements (TEs) of many different types (Additional file 1: Table S3) the majority of which are *copia*- and *gypsy*-like retrotransposons (12,604 bp). Most TEs were identified in intergenic regions (Figure 2) with only 1,095 bp (6.8%) inserted into genic regions (Additional file 1: Table S4).

#### Open reading frames

One hundred twenty-three mitochondrial ORFs (≥300 bp in length) were identified in *Rhazya* intergenic regions (Additional file 1: Table S5). Blast searches against the non-redundant nucleotide and protein databases in the NCBI identified several ORFs with high similarity to hypothetical proteins, some of which were cytoplasmic male sterile (CMS) related ORFs, or were derived from intergenic regions of other plant mitochondrial genomes. Other ORFs contained sequences similar to organellar or viral DNA and RNA polymerases, retrotransposons or nuclear genes. The remaining ORFs had no significant similarity to any genes or proteins in the NCBI database.

BlastN queries of the *Rhazya* transcriptome assembly (subject database) detected transcripts for many of the 123 ORFs (Additional file 1: Table S5). One transcript had a sequence identical to a mitochondrial copy of a nuclear gene, (R)-mandelonitrile lyase, which is present as split ORFs in the mitochondrial genome of *Rhazya* due to a 5 bp deletion relative to the nuclear gene (Additional file 2: Figure S3). RT-PCR confirmed that the transcript has 100% sequence identity to the mitochondrial DNA sequence, including the nonsense mutation.

Twelve ORFs of at least 150 bp in length, three of which are present as two copies, appeared to be chimeric ORFs that contain small fragments (>30 bp) of mitochondrial genes (Additional file 1: Table S6). Five of these ORFs were predicted to encode one or two transmembrane helices. ORF56b contains small fragments of the three mitochondrial genes, *rpl2*, *matR* and *ccmFn*, that overlap with repeat 6 (Additional file 2: Figure S4A). Two ORFs are located in the region with three or four repeats that are associated with *atp6* (Additional file 2: Figure S4B). While each of these two were predicted to encode a protein with two transmembrane helices, neither could be identified by a BlastN search against annotated *Rhazya* mitochondrial genes.

#### Sequences of plastid origin

Plastid-like sequences were found in 38 fragments throughout the *Rhazya* mitochondrial genome, ranging from 75 to 5,069 bp in length (Figure 2; Additional file 1: Table S7). The total amount of plastid sequence was 32,810 bp, representing 6.0% of the mitochondrial genome. The 38 insertion regions represent 24.1% of the *Rhazya* plastid genome. Six intact plastid genes, *ndhH*, *atpH*, *psaB* (*x*2), *psaA* (*x*2), eight tRNAs (one of which had two copies), six pseudogenes and numerous partial genes and intergenic spacer regions were identified. All plastid fragments were located in intergenic regions. Among the tRNAs, four differed by one to three nucleotide substitutions from copies in the plastid (one *trnD-GUC*, two *trnH-GUG*, two *trnS-GGA*, and three *trnN-GUU*). There are two degenerate copies of *trnI-CAU* and *trnP-UGG* with six indels compared to the plastid-encoded tRNAs. Plastid-derived *trnM-CAU* was not detected in the *Rhazya* mitochondrial genome.

#### Mitochondrial RNA editing

PREP-Mt and PREPACT predicted 462 and 480 putative C-to-U RNA editing sites, respectively, in the 38 *Rhayza* mitochondrial protein-coding genes (Additional file 1: Table S8). Cytochrome c proteins and NADH dehydrogenase subunits (complex I) were more highly edited than other protein-coding genes, whereas ribosomal proteins had fewer edited sites. Available transcriptome data for 11 genes (*atp1*, *atp9*, *cox1*, *cox2*, *cox3*, *nad4*, *nad5*, *nad7*, *rpl5*, *rps4*, *rps7*) confirmed that of the 157 sites predicted by PREP-Mt for these genes, 148 (96%) sites were edited (Additional file 1: Table S9). In the *Rhazya* mitochondrial genome, *atp6* and *rps10* begin with an ACG start codon instead of the standard ATG. In the case of *rps10*, PREP-MT predicted that the start codon was altered by RNA editing (score 1.0) to the standard ATG codon, whereas the editing prediction for *atp6* was only 0.25. RNA editing was also predicted to generate the stop codons in *atp6*, *atp9*, and *rps10* (score 1.0).

### Characterization of putative functional gene transfers to the nucleus

**Figure 3:**
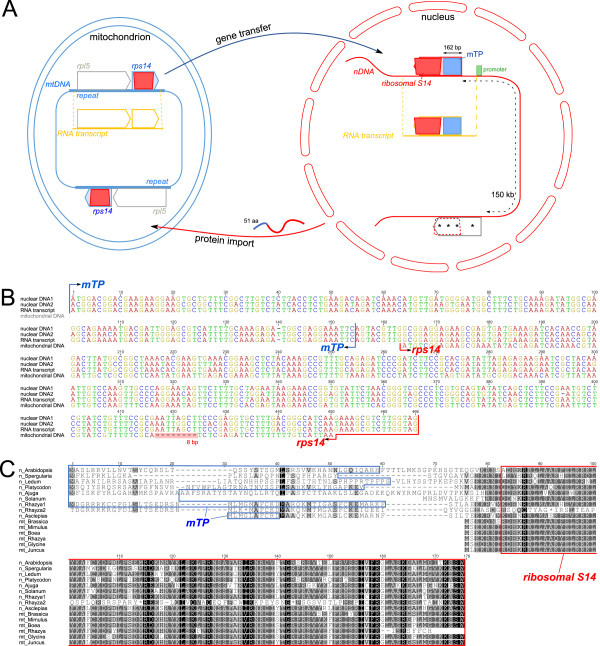
**Gene transfer of** ***rps14*** **. A**. Schematic diagram of *rps14* gene transfer from mitochondrial genome to the nucleus. The mitochondrial *rps14* copies are identified in two repeat regions and are co-transcribed with *rpl5*. Boxes indicate mitochondrial targeting presequence (mTP; blue) and a conserved domain (*ribosomal S14*; red). The grey box arrow in the nuclear genome represents the non-functional copy of *rps14* and the dotted red line indicates a conserved domain. Internal stop codons are indicated with asterisks. **B**. Nucleotide sequence alignment of the two nuclear, transcript, and mitochondrial copies of *rps14* from *Rhazya stricta*. Shaded red box shows an 8 bp deletion that caused a frameshift. **C**. Amino acid sequence alignment of two nuclear and one mitochondrial copy of *rps14* of *Rhazya* with seven nuclear-encoded and five mitochondrial copies from other angiosperms (see Additional file 1: Table S13). Blue boxes indicate mitochondrial targeting presequences. Red boxes indicate the conserved domain of ribosomal S14. mt = mitochondrial, n = nuclear.

**Figure 4:**
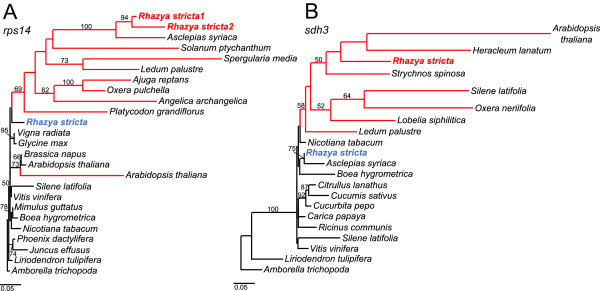
**Maximum likelihood phylogenetic trees for mitochondrial genes of** ***Rhazya*** **transferred to the nucleus. A**. Mitochondrial and nuclear *rps14* sequences of angiosperms. **B**. Mitochondrial and nuclear *sdh3* sequences of angiosperms. Bold font indicates mitochondrial (blue) and nuclear copies (red) of *rps14* and *sdh3* in *Rhazya stricta*. Bootstrap support values >50% are shown at nodes. Red and black lines indicate nuclear and mitochondrial sequences, respectively.

All 38 protein-coding genes in the *Rhazya* mitochondrial genome were used to query the *Rhazya* transcriptome assembly (133,266 contigs). An ORF with 77.7% nucleotide sequence identity to the *rps14* gene was identified that includes a 5’ extension of 162 bp (Figure 3A and 3B). The first 51 amino acids of this ORF were predicted by TargetP to be an mTP (mitochondrial = 0.6), whereas Predotar returned the prediction of ‘elsewhere’ (elsewhere = 1.0). Examination of a draft *R. stricta de novo* nuclear genome sequence (D. Arasappan, unpublished) confirmed the presence of an intronless, nuclear-encoded *rps14* gene showing 100% nucleotide sequence identity to the transcript (Figure 3B). Another copy of *rps14* with 90.6% nucleotide sequence identity was detected on the same scaffold approximately 150 kb from the first copy, however it lacks a proximal start codon and has four internal stop codons (Figure 3A and 3C). Phylogenetic analysis of nuclear and mitochondrial copies of *rps14* showed that the *Rhazya* mitochondrial copy did not group with other asterids but instead was positioned sister to the clade that includes nuclear copies from other angiosperms (Figure 4A). However, support for this placement was very weak (<50% bootstrap value) and resolution of relationships among the mitochondrial copies was low. The two nuclear copies of *R. stricta* grouped together with high bootstrap support (94%), and branch lengths on the tree indicate that the putative non-functional copy (i.e. the one lacking an mTP) has experienced much higher substitution rates (*Rhazya stricta*2 in Figure 4A). Protein sequence alignment of the extended amino acids upstream from *rps14* was highly divergent with very low identities between *Rhazya* and seven other species, ranging from 12.5% to 75.8% (Figure 3C). Protein sequence alignment of the nuclear copy of *Rhazya rps14* to mitochondrial-encoded sequences from five species of seed plants showed pairwise identities ranging from 73.1% to 75.3%, higher than the pairwise identity between the nuclear and mitochondrial *Rhazya rps14* copies at 65.5% (Figure 3C).

**Figure 5:**
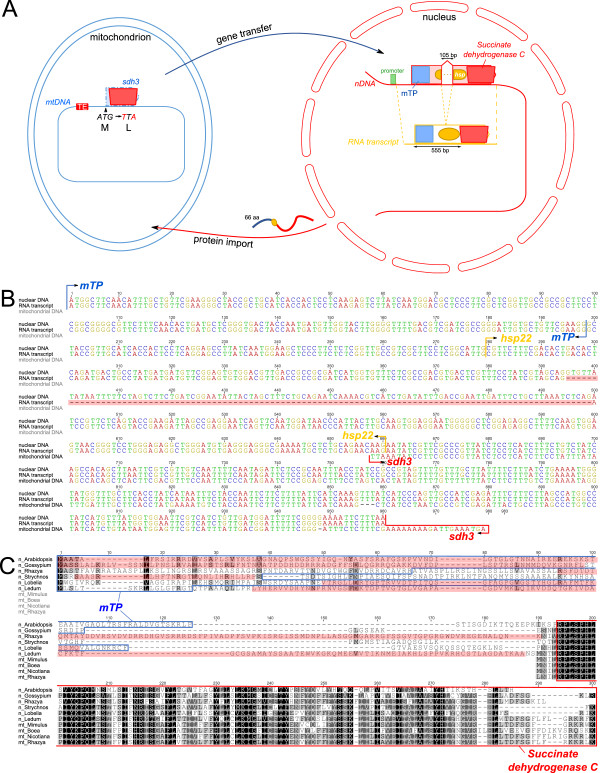
**Gene transfer of** ***sdh3*** **. A**. Schematic diagram of *sdh3* gene transfer from mitochondrial genome to the nucleus. A transposable element (TE) insertion is shown in red in the mitochondrial DNA. The dashed line outlines a pseudogene due to mutations in the start codon as indicated. Boxes and oval indicate mitochondrial targeting presequence (mTP; blue), heat shock protein (*hsp*; orange), and a conserved domain (*succinate dehydrogenase C*; red). **B**. Nucleotide sequence alignment of the nuclear, transcript, and mitochondrial copies of *Rhazya stricta*. The shaded red area shows the intron within predicted heat shock protein (*hsp22*). **C**. Amino acid sequence alignment of *Rhazya* nuclear and mitochondrial *sdh3* with six nuclear-encoded and three mitochondrial-encoded copies from other angiosperms (see Additional file 1: Table S13). Boxes indicate mitochondrial targeting presequence (blue) and a conserved domain of succinate dehydrogenase C (red), and shaded red boxes indicate the remaining portions of genes into which *sdh3* was transferred (heat shock proteins, *Rhazya* and *Gossypium*-*hsp22*, *Arabidopsis*-*hsp70;* Iron-sulfur cluster scaffold-like protein, *Ledum*; hypervariable *Bacillus* group-specific protein, *Strychnos*; Pyrimidine (PYR) binding domain of thiamine pyrophosphate (TPP)-dependent enzyme, *Lobelia*). mt = mitochondrial, n = nuclear.

The *Rhazya* transcriptome assembly was queried with the *sdh3* pseudogene sequence from the mitochondrial genome. A contig containing an *sdh3*-like ORF with 81.5% nucleotide sequence identity to the query sequence was detected (Figure 5A and 5B). The ORF included a 5’ extension of 555 bp and the first 66 amino acids of the ORF were predicted by TargetP (0.8) and Predotar (0.9) to be an mTP. Phytozome predicted a small heat shock protein (*hsp22*) between the targeting presequence and the *sdh3* coding region (Figure 5B and 5C). Examination of the draft *R. stricta de novo* nuclear genome assembly confirmed the presence of the nuclear-encoded *sdh3* gene containing two exons totaling 960 bp separated by a 105 bp intron (Figure 5A and 5B). Phylogenetic analysis of nuclear and mitochondrial copies of *sdh3* showed that the *Rhazya* mitochondrial copy was nested within a clade of asterid mitochondrial copies and the nuclear-encoded copy grouped within a nuclear-encoded clade (Figure 4B). Protein sequence alignment of the predicted targeting presequence and the remaining sequence upstream from *sdh3* was highly divergent with low amino acid identities between *Rhazya* and five other species, ranging from 12.2% to 22.2% (Figure 5C). Protein sequence alignment of the nuclear copy of *Rhazya sdh3* to nuclear- and mitochondrial-encoded sequences from several species revealed low amino acid identities of 50.0-59.4% and 46.4-54.9%, respectively (Figure 5C).

### Comparison of *Rhazya* mitochondrial genome to seven other asterids

**Figure 6:**
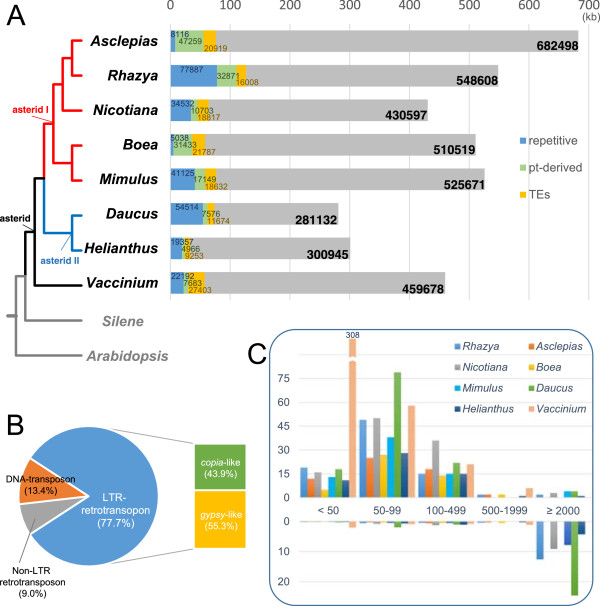
**Genome size, amount of plastid-like and repetitive DNA and transposable elements in eight asterid mitochondrial genomes. A**. Genome sizes, the number of bp of repetitive DNA, plastid-derived sequences and transposable elements. See Figure 8 legend for details of how the tree was constructed. **B**. Average of the percentage of different types of transposable elements of asterids. **C**. Repeat size and frequency (above), and proportion of repetitive DNA per genome (below).

Mitochondrial genome sizes of the eight asterids range from 281,132 bp in *Daucus* to 682,498 bp in *Asclepias* (Figure 6A; Additional file 1: Table S10). Using BlastN searches (e-value 1e-6), *Rhazya* was found to share more sequences (214 kb) with *Asclepias*, both coding and non-coding, than with other asterids (*Nicotiana* - 171 kb, *Mimulus* - 168 kb, *Boea* - 146 kb, *Daucus* - 148 kb, *Helianthus* - 125 kb, and *Vaccinium* - 128 kb). GC content across the eight genomes ranges from 43.3% in *Boea* to 45.4% in *Daucus* (Additional file 1: Table S10). Only one colinear gene block was identified among the eight asterid genomes: *rrn18-rrn5* (Additional file 2: Figure S5).

BlastN searches of each mitochondrial genome against its plastid counterpart revealed that plastid derived sequences account for 2.5-6.9% of the eight asterid mitochondrial genomes (Figure 6A; Additional file 1: Table S10). The number of tRNAs derived from plastids varies among the eight asterids, ranging from five in *Daucus* to 11 in *Boea* (Additional file 1: Table S11). All asterids examined share four (*trnD-GUC*, *trnH-GUG*, *trnN-GUU*, and *trnW-CCA*) intact plastid-derived tRNAs. In addition to plastid-derived sequences, the eight asterid mitochondrial genomes contain 2.9-6.0% TEs (Figure 6A; Additional file 1: Table S10), most of which were LTR-retrotransposons (Figure 6B). The percentage of TEs in *Rhazya* is the lowest among the asterids examined.

**Figure 7:**
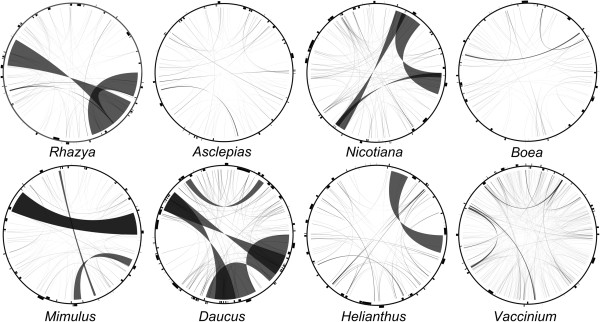
**Distribution of repetitive DNA in** ***Rhazya*** **mitochondrial genome compared to seven other asterids**. Black lines within circular maps indicate the positions of the pairs of repeats, with crossed connecting lines denoting reverse repeats. Black boxes on the inner and outer circle indicate the positions of mitochondrial genes.

Repetitive DNA content among asterid mitochondrial genomes is highly variable, ranging from 1.0% in *Boea* to 19.4% in *Daucus* (Figure 6A; Additional file 1: Table S10). All asterids had numerous small repetitive DNAs (<100 bp), with the greatest number found in *Vaccinium* (Figure 6C; Additional file 1: Table S10). The mitochondrial genome of *Rhazya* contains longer repeats, whereas *Daucus* had a higher percentage of repetitive DNA. Repetitive DNA is widely scattered across the eight asterid genomes, and each genome contains a unique pattern of repeats (Figure 7).

**Figure 8:**
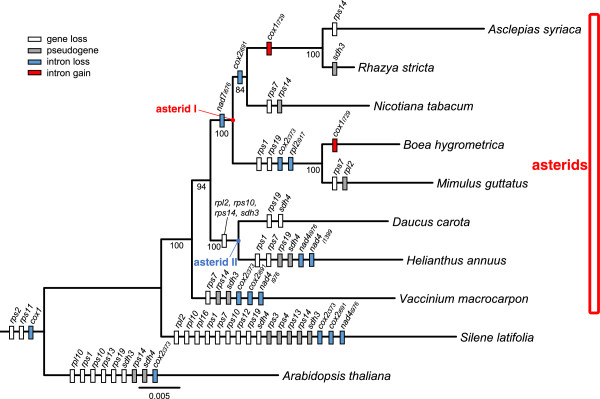
**Phylogenetic distribution of gene/intron content (loss and gain) among eight asterids and two outgroups**. The tree was inferred using the maximum likelihood (ML) method with the ‘GTRGAMMA’ evolutionary model under the rapid bootstrap algorithm (1000 replicates). Bootstrap support values > 50% are shown at nodes. The 24 genes include *atp[1,4,6,8,9]*, *ccm[B, C, Fc, Fn]*, *cob*, *cox[1-3]*, *matR*, *mttB*, and *nad[1, 2, 3, 4, 4L, 5, 6, 7, 9]*. The *cox1* introns have been lost in the ancestor of angiosperms.

The phylogenetic distribution of genes and introns among the eight asterids and one representative species each from Caryophyllales and rosids is shown in Figure 8. This analysis revealed a number of shared and unique gene and intron gains/losses among asterids. The asterid mitochondrial genomes share 31 protein-coding, 3 rRNA and 12 tRNAs, however gene and intron content across asterids varies considerably for the other protein genes (Tables 2 and 3; Additional file 1: Table S11).

**Table 2:** ***Rhazya*** **protein-coding gene content compared to other asterids and two representative angiosperms**

	***Rhazya***	***Asclepias***	***Nicotiana***	***Mimulus***	***Boea***	***Daucus***	***Helianthus***	***Vaccinium***	***Silene***	***Arabidopsis***
*atp[x]*	●	●^a^	●	●	●	●	●	●^d^	●	●
*ccm[x]*	●	●	●	●	●	●	●	●	●	●
*cob*	●	●	●	●	●	●	●	●^d^	●	●
*cox[x]*	●	●	●	●	●	●	●^c^	●	●	●
*matR*	●	●	●	●	●	●	●	●	●	●
*mttB*	●	●	●	●	●^b^	●	●^c^	●	●	●
*nad[x]*	●	●	●	●	●	●	●^c^	●^d^	●	●
*rpl2*	●	●	●	ψ	●^b^	○	○	●	○	●
*rpl5*	●	●	●	●	●^b^	●	●	●	●	●
*rpl10*	●	●	●	●	●^b^	●	●	●	○	○
*rpl16*	●	●^a^	●	●	●	●	●	●	○	●
*rps1*	●	●	●	○	○	●	○	●	○	○
*rps2*	○	○	○	○	○	○	○	○	○	○
*rps3*	●	●	●	●	●	●	●^c^	●^d^	ψ	●
*rps4*	●	●	●	●	●	●	●	●	ψ	●
*rps7*	●	●	○	○	●	●	○	○	○	●
*rps10*	●	●	●	●	●^b^	○	○	●	○	○
*rps11*	○	○	○	○	○	○	○	○	○	○
*rps12*	●	●	●	●	●	●	●	●	○	●
*rps13*	●	●	●	●	●	●	●	●	ψ	○
*rps14*	●	○	ψ	●	●^b^	○	○	ψ	ψ	ψ
*rps19*	●	●	●	○	○	○	ψ^c^	●	○	○
*sdh3*	ψ	●	●	●	●	○	○	ψ	ψ	○
*sdh4*	●	●	●	●	●	○	ψ^c^	●	○	ψ
● present	38	38	37	35	37	33	31	36	25	31
○ absent	2	3	3	5	4	8	8	3	11	8
Ψ pseudo	1	0	1	1	0	0	2	2	5	2

^a^Although *atp6* and *rpl16* of *Asclepias syriaca* are annotated in GenBank as pseudogenes, new analyses suggest functionality for both genes. ^b^ *Boea hygrometrica*, ^c^ *Helianthus annuus,* and ^*d*^ *Vaccinium macrocarpon* mitochondrial genomes were re-annotated using MITOFY [27] in this study. *atp[x]* = *atp*[*1,4,6,8,9*]*, ccm[x] = ccm[B, C, Fc, Fn], cox[x] = cox*[*1-3*], *nad[x]* = *nad[1, 2, 3, 4, 4L, 5, 6, 7, 9]*.

**Table 3:** ***Rhazya*** **intron content compared to other asterids and two representative angiosperms**

	***Rhazya***	***Asclepias***	***Nicotiana***	***Mimulus***	***Boea***	***Daucus***	***Helianthus***	***Vaccinium***	***Silene***	***Arabidopsis***
*ccmFci829*	●	●	●	●	●	●	●^b^	●	●	●
*cox1i729*	●	●	○	○	●	○	○	○	○	○
*cox2i373*	●	●	●	○	○	●	●^b^	○	○	○
*cox2i691*	○	○	○	●	●	●	●^b^	○	○	●
*nad1i394*	□	□	□	□	□	□	□^b^	□	□	□
*nad1i477*	●	●	●	●	●	●	●^b^	●	●	●
*nad1i669*	□	□	□	□	□	□	□^b^	□	□	□
*nad1i728*	□	□	□	□	□	□	□^b^	□	□	●
*nad2i156*	●	●	●	●	●	●	●^b^	●	●	●
*nad2i542*	□	□	□	□	□	□	□^b^	□	□	□
*nad2i709*	●	●	●	●	●	●	●^b^	●	●	●
*nad2i1282*	●	●	●	●	●	●	●^b^	●	●	●
*nad4i461*	●	●	●	●	●	●	●^b^	●	●	●
*nad4i976*	●	●	●	●	●	●	○^b^	○	○	●
*nad4i1399*	●	●	●	●	●	●	○^b^	●	●	●
*nad5i230*	●	●	●	●	●	●	●^b^	●	●	●
*nad5i1455*	□	□	□	□	□	□	□^b^	□	□	□
*nad5i1477*	□	□	□	□	□	□	□^b^	□	□	□
*nad5i1872*	●	●	●	●	●	●	●^b^	●	●	●
*nad7i140*	●	●	●	●	●	●	●^b^	●	●	●
*nad7i209*	●	●	●	●	●	●	●^b^	●	●	●
*nad7i676*	○	○	○	○	○	●	●^b^	●	●	●
*nad7i917*	●	●	●	●	●	●	●^b^	●	●	●
*rpl2i917*	●	●	●	○	○^a^	x	x	●	x	●
*rps10i235*	●	●	●	●	●^a^	x	x	○	x	x
*rps3i74*	●	●	●	●	●	●	●^b^	●^c^	●	●

●; *cis*-spliced, □; *trans*-spliced, ○; intron absent, x; gene loss. The genes of ^a^ *Boea hygrometrica* and ^b^ *Helianthus annuus* and ^c^ *Vaccinium macrocarpon* mitochondrial genomes were re-annotated using MITOFY [27] in this study.

All species in the asterid I lineage were missing the intron *nad7i676. Rhazya, Asclepias* and *Nicotiana* share the loss of the intron *cox2i691*, and *Boea* and *Mimulus* share the loss of two genes (*rps1* and *rps19*) and two introns (*cox2i373* and *rpl2i917*). The phylogenetic distribution of the intron *cox1i729* (Figure 8) indicates that the gain of this intron occurred independently in the *Rhazya/Asclepias* clade and *Boea*. Among the asterid I species analyzed, the presence of *sdh3* as a pseudogene was unique to *Rhazya*. Two species, *Helianthus* and *Vaccinium*, in the asterid II lineage share the loss of four genes (*rpl2*, *rps10*, *rps14*, and *sdh3*). No shared losses/gains were found for the asterid clade relative to the two outgroups.

BlastN searches of the (R)-mandelonitrile lyase gene, which is represented by two overlapping ORFs in *Rhazya* (Additional file 2: Figure S3), were performed against asterid mitochondrial genomes. Six additional asterids, *Asclepias* (66 bp), *Boea* (193 bp), *Daucus* (520 bp), *Helianthus* (273 bp), *Nicotiana* (495 bp), and *Vaccinium* (246 bp) contain fragments of this nuclear gene (Additional file 2: Figure S3B).

## Discussion

### Organization of *Rhazya stricta* organelle genomes

The *R. stricta* plastid genome is highly conserved with gene content and gene order identical to the ancestral genome organization of angiosperms [14, 37]. The size of *R. stricta* plastid genome at 154,841 bp is close to the median genome size for photosynthetic land plants [38]. A recent study found that the plastid genome of *Asclepias* contains a 2.4 kb segment of mitochondrial DNA with an *rpl2* pseudogene, and this transfer has been documented in other genera of the tribe Asclepiadeae (subfamily Asclepiadoideae, Apocynaceae) [39]. The *Rhazya* plastid genome does not contain any mitochondrial-like sequences, confirming that transfer of mitochondrial DNA into the plastid genome is restricted to a single tribe of Apocynaceae. Therefore, inter-compartmental transfers between the *Rhazya* plastid and mitochondrial genomes have occurred in one direction only.

The *R. stricta* mitochondrial genome exhibits several complex evolutionary features, including a dynamic genome structure that has been shaped by repeat families and intramolecular recombination, invasion of the genome by both plastid and nuclear sequences, and the putative functional transfer of two genes to the nucleus. Recombination between repeats has resulted in a master chromosome and multiple subgenomic circles [19–22, 40]. The *R. stricta* mitochondrial genome contains seven repeat families, including two large and five intermediate-sized repeats. These repeats are involved in homologous recombination in the *Rhazya* mitochondrial genome. Two distinct conformations of the master chromosome and six subgenomic circles have been confirmed by PCR (Additional file 2: Figures S1 and S2), however additional chromosome configurations may be present in *Rhazya* mitochondria. PCR recombination has been suggested as a complicating factor in utilizing this approach to confirm recombination among repeats in plant mitochondrial genomes [21]. However, the use of three different Illumina libraries (one paired end and two mate pair), the high depth of coverage (average coverage is 679X), and corrected PacBio data provide additional confidence in the PCR results. Recombination among the repeats may have influenced gene content by facilitating gene fusion and pseudogenization events [41]. For example, most mitochondrial genes of *Rhazya* are highly conserved in length compared to other angiosperms except *atp6*. This gene usually contains extended sequences at the N-terminus that are highly divergent across angiosperms even within species [42]. In most cases, *atp6* is located at the border of repeat regions, suggesting that this gene is frequently involved in genomic recombination [42]. In *Rhazya* only the *atp6* conserved domain is present and is located downstream from repeat 2 (R2; Additional file 2: Figure S4B). The N-terminal extended sequences of *atp6* in *Rhazya* may have been cleaved by genomic recombination activities upstream of the protein gene, but remnants of these extended sequences were not detected due to high sequence divergence and frequent recombination activities. Two ORFs (318 and 324) located within repeat families including repeat 2 have one of the diagnostic features of chimeric genes: the presence of a transmembrane domain [24] (Additional file 2: Figure S4B). Studies have shown that chimeric ORFs containing a portion of ATP synthase subunit are frequently associated with rearrangements in plant mitochondrial genomes [22, 24]. The situation in *Rhazya* supports a connection between ATP synthase subunits and recombination in mitochondrial genomes.

Twelve other potential chimeric ORFs are present in the *R. stricta* mitochondrial genome. One of these, ORF56b, has features of CMS [24], including fragments containing a portion of three mitochondrial genes (*rpl2*, *matR,* and *ccmFn*), transmembrane helices and overlap with a second copy of repeat 6 (R6; Additional file 2: Figure S4A). Further work is needed to determine if these other ORFs are functional and associated with genomic recombination.

Plant mitochondrial genomes typically contain DNA originating from plastid and nuclear genomes, and in some cases from other species including bacteria, viruses and plants [25–29]. The *Rhazya* mitochondrial genome contains considerable foreign DNA, accounting for at least 8.9% of the genome. Plastid-derived sequences are variable in mitochondrial genomes of seed plants and account for 1-12% [17] indicating that *Rhazya* has an intermediate amount in its genome (6%). The inserted plastid sequences of *Rhazya* include full-length protein-coding and tRNA genes, most of which are presumed nonfunctional, but some tRNA genes may be candidates for functional transfer as reported for wheat and potato [43, 44]. The absence of *trnM-CAU* in *Rhazya* is unusual among sequenced angiosperm mitochondrial genomes as previous phylogenetic analyses have suggested that transfer of *trnM-CAU* to the mitochondrion occurred in the common ancestor of extant angiosperms [45, 46].

The *Rhazya* mitochondrial genome also contains numerous nuclear-derived sequences, most of which are transposable elements (TEs) (Figure 6A and 6B; Additional file 1: Table S3). Most of the TEs are located in intergenic spacers but 14 are found in genes (Additional file 1: Table S4). Previous studies have only observed TEs in intergenic spacers reviewed in [17] but in some cases searches for TEs were limited to these regions see reference [27]. In addition to TEs, the *Rhazya* mitochondrial genome contains two sequences homologous to the nuclear gene encoding (R)-mandelonitrile lyase. One copy of the sequence is nearly complete and the presence of a transcript with 100% sequence identity to this copy suggests that it is expressed. Previous studies have shown that some angiosperm mitochondrial genomes contain fragments of nuclear protein-coding genes [21, 27, 47], however the assembled transcriptome contig and RT-PCR result refute a functional role for this gene in *Rhazya* mitochondria. The presence of internal stop codons in the transcript suggests that it is likely a product of relaxed transcription in mitochondria [48].

The *Rhazya* mitochondrial genome has acquired the intron *cox1i729*, which is commonly the subject of horizontal transfer across angiosperms [49]. This intron in *Rhazya* is highly similar (96.7-98.8% sequence identity) to introns from eight other genera of Apocynaceae. Sanchez-Puerta et al. [49] suggested six independent gains of this intron in the Apocynaceae. Two alternative hypotheses for the *cox1* intron were suggested: stochastic loss and horizontal transfer [50, 51]. The dynamics of *cox1* intron gain or loss within the Apocynaceae requires further study to better understand its evolutionary history in the family.

### Mitochondrial gene transfer in *Rhazya* genomes

*Rhazya* contains 38 of the 41 protein-coding genes that are found in the ancestral angiosperm mitochondrial genome [17]. Two ribosomal proteins, *rps2* and *rps11*, that are absent in *Rhazya* have been lost from the mitochondrial genomes of nearly all core eudicots [52]. The third gene, *sdh3*, has been lost numerous times across angiosperms [52] and appears to be a pseudogene in *Rhazya*. Analyses of the transcriptome and a draft nuclear genome of *Rhazya* facilitated identification of putative functional copies of *sdh3* and *rps14* in the nucleus.

*Rhazya sdh3* has been transferred to the nucleus and has acquired a mitochondrial targeting presequence as well as one intron and a portion of chaperonin gene *hsp22* (Figure 5). Previous studies have identified 14 independent transfers of *sdh3* to the nucleus in angiosperms, and in all cases mitochondrial targeting presequences were acquired from preexisting nuclear genes [36, 53]. A previous study reported putative functional transfers of *sdh3* in five other asterid families, Asteraceae, Convolvulaceae, Ericaceae, Lamiaceae and Orobanchaceae [36]. This previous report, combined with the phylogenetic distribution of *sdh3* loss from the mitochondrial genomes of sequenced asterid genomes (Figure 8), indicates that this gene has been transferred to the nucleus multiple times in this clade.

In case of the *Rhazya rps14*, two copies were identified in the nucleus. One copy has a mitochondrial targeting presequence and is likely functional while the second copy is likely non-functional considering its level of divergence from the putative functional copy (Figures 3 and 4A). There are at least three alternative explanations for the origin of two nuclear copies: 1) *rps14* was transferred to the nucleus and after acquiring the mitochondrial targeting presequence it was duplicated, followed by the loss of the targeting presequence in one copy; 2) the gene was duplicated after transfer and only one copy acquired a targeting presequence; or 3) there were two independent transfers of *rps14* to the nucleus and only one of the copies acquired a targeting presequence. Phylogenetic analyses support a single transfer of *rps14* to the nucleus (Figure 4A) but it is not possible with available data to discern between alternatives one and two. A previous study showed that the *rps14* coding sequence has been transferred to the nucleus independently at least three times in grasses and that mitochondrial *rps14* pseudogene transcripts are expressed [54], although this may be due to the well-known phenomenon of relaxed transcription in mitochondria [48]. Although *Rhazya* mitochondrial *rps14* has a deletion at the 3’ end causing a frameshift mutation, the gene may retain functionality as it is nearly full length relative to other angiosperms (Figure 3B and 3C). Moreover, the mitochondrial copy lacks nonsense mutations and transcriptome data showed that the mitochondrial *rps14* is co-transcribed with *rpl5* (Figure 3A) as a reported for tobacco [19]. Following transfers to the nucleus, the co-existence of putative functional nuclear and mitochondrial gene copies has been suggested for only three genes among angiosperms, including *cox2* in some Fabaceae [55], *rpl5* in *Triticum*[56], and *sdh4* in *Populus*[57].

### Evolutionary comparisons among asterid mitochondrial genomes

Comparing the mitochondrial genome sequences of *Rhazya* with the seven other asterids and two representative non-asterids provides insight into the distinct evolutionary events that have occurred across this clade. Genome sizes vary 2.4-fold among the asterids and the *Rhazya* genome represents an intermediate size within the asterid I lineage (Figure 6A). No clear correlation is seen between repetitive DNA or TE content and genome size among asterid mitochondrial genomes, whereas the amount of transferred plastid DNA tends to scale with genome size. For example, repetitive DNA content is highest in *Rhazya*, but *Asclepias* is the largest genome despite its 9.6-fold lower repetitive DNA content than *Rhazya* (Figure 6A). Likewise, although *Daucus* has the second highest repetitive DNA content, it is the smallest genome among the eight asterids (Figure 6A). The asterid mitochondrial genomes also differ in the structure and complexity of their repeats. *Rhazya*, *Nicotiana, Mimulus*, *Helianthu*s and *Daucus* have large repeats, in contrast to the other asterids. These genomes display diverse patterns of repeats that appear to facilitate recombination and can range in size from short (124 bp) to long (36 kb) within a single species.

There are very few colinear clusters of genes across the eight asterid mitochondrial genomes, which likely reflects the very different patterns of repeats that have caused rearrangements (Figure 7). Overall, this comparison indicates that asterid mitochondrial genomes have contrasting evolutionary histories, resulting in very diverse organization and gene content. A better understanding of the evolutionary history of recombination among asterid mitochondrial genomes requires genome sequences for more taxa and comparisons of nuclear genes encoding DNA repair and recombination proteins including RecA-like recombinases, MutS homologue 1, the Whirlies and other organellar single stranded DNA-binding proteins that have been implicated in maintenance of genome stability [58].

The phylogenetic distribution of gene and intron losses and TE insertions revealed some shared and many independent events across asterids (Figure 8, Additional file 1: Table S4). Most mitochondrial gene loss/pseudogenization events involve ribosomal proteins and *sdh* genes, as in other angiosperms [52]. The two protein-coding genes *rps2* and *rps11* were lost in the ancestor of eudicots [52]. The presence of the intron *cox1i729* in the *Rhazya/Asclepias* clade and *Boea* suggests that it was gained independently twice in the asterid clade since this intron was lost in the ancestor of angiosperms [49]. The asterid I lineage shares the loss of the intron *nad7i676*, indicating that this loss occurred in the common ancestor of the clade. The loss of the intron *cox2i691* in *Rhazya*, *Asclepias* and *Nicotiana*, and the loss of two genes (*rps1* and *rps19*) and two introns (*cox2i373* and *rpl2i917*) in *Boea* and *Mimulus* indicate that these events occurred in the most recent common ancestor of each clade. In case of the *Mimulus rpl2*, the phylogenetic distribution suggests that this gene became pseudogenized after the intron was lost in common ancestor of the *Boea*/*Mimulus* clade. The asterid II clade has lost four genes, three ribosomal proteins genes and one *sdh* gene. The phylogenetic distribution of *rps14* among asterids suggests that this gene was transferred to the nucleus in the common ancestor of the *Rhazya*, *Asclepias* and *Nicotiana* clade. The fate of the gene was different in the three species; it has been lost in *Asclepias*, pseudogenized in *Nicotiana* while both the mitochondrial and nuclear copies are likely functional in *Rhazya*. The duplication event of nuclear *rps14* within the asterid I clade is likely more complicated and will require more nuclear genome data to resolve. There are a number of shared gains of TEs within genic regions across asterids (Additional file 1: Table S4). All asterids examined share the same TEs in the same location except for the TE in the *cox3* gene. This suggests that nearly all TEs were transferred in the ancestor of the asterid clade. The *cox3* TE insertion in *Rhazya* and *Nicotiana* differ in size (86 versus 41 bp) and TE class (LTR/Copia in *Rhazya* and DNA/MuDR in *Nicotiana*).

## Conclusions

Organelle genomes of *Rhazya stricta*, a member of the asterid I clade, provide important information for improving the understanding of mitochondrial genome evolution among angiosperms. The mitochondrial genome exhibits a number of phenomena that have been observed in other species [17], including the presence of recombinogenic repeats that generate a multipartite organization with a master chromosome and subgenomic circles, a high incidence of transferred DNA from the plastid and nuclear genomes, and gene transfers from the mitochondrion to the nucleus. The organellar genomic sequences, combined with nuclear transcriptome and genome data, have enabled a rigorous examination of these events. *Rhazya* is unique among the eight sequenced asterids in the types of events that have shaped the evolution of its mitochondrial genome. The organelle genomes of *R. stricta* provide valuable genomic resources for utilizing this important medicinal plant in biotechnology applications.

## Methods

### Plant material

*Rhazya stricta* seeds were obtained from natural populations collected in the Makkah Province, Saudi Arabia. Seeds were soaked in water overnight at 37°C then transferred to Profile® Field & Fairway™ inorganic ceramic particles (Buffalo Grove, IL) in a growth chamber (16 h light, 8 h dark, 38°C) for germination. Young leaves were flash frozen in liquid nitrogen for DNA and RNA isolation and stored at -80°C.

### DNA isolation

Genomic DNA isolation was performed as described by Doyle and Doyle [59] with modifications. Cetyl trimethylammonium bromide buffer was augmented with 3% polyvinylpyrrolidone and 3% beta-mercaptoethanol (Sigma, St. Louis, MO). Organic phase separation was repeated until the aqueous fraction was clear. DNA pellets were resuspended in ~200 μL DNase-free water. Following treatment with RNase A (ThermoScientific, Lafayette, CO) samples were again subjected to phase separation with chloroform, and DNA was recovered by ethanol precipitation. Samples were resuspended in DNase-free water, evaluated for intactness and concentration by gel electrophoresis and stored at -20°C.

### DNA sequencing and genome assembly

Genomic DNA was used to construct three Illumina libraries: a paired-end (PE) fragment library with an average insert size of 626 bp, and two mate pair (MP) libraries with an average insert sizes of 2,363 and 4,340 bp. Sequence reads were generated using the HiSeq 2000 sequencing platform (Illumina, San Diego, CA) at the Genome and Sequence Analysis Facility (GSAF) at the University of Texas at Austin. A 10 kb SMRT cell library was constructed for PacBio RS II sequencing (Pacific Biosciences, Menlo Park, CA) and eight SMRT cells of sequence data were generated at the University of Florida Interdisciplinary Center for Biotechnology Research. All PacBio reads were corrected using PacBioToCA [60] with 634 Mb (10%) of PE Illumina reads.

To complete the plastid genome, the PE Illumina reads were assembled *de novo* with Velvet v.1.2.08 [61] using multiple *k*-mers. For the mitochondrial genome, Illumina reads were assembled *de novo* with Velvet using multiple *k*-mers values and by separately combining the PE reads with each of the two MP read sets. The parameters were modified according to the depth of the read coverage of each organelle genome. The initial plastid contigs were assembled in Geneious R6 v.6.1.6 [62], which was also used to visualize and finish the mitochondrial genome assembly by tracking and end inspection of the initial mitochondrial contigs overlapping with corrected PacBio reads. The corrected PacBio reads were further used to fill gaps and to validate regions of plastid integration in the mitochondrial genome. The correction of PacBio reads and the *de novo* genome assemblies were performed on Lonestar Dell Linux Cluster of the Texas Advanced Computing Center (TACC).

### Genome annotations and analyses

The plastid and mitochondrial genomes were annotated using DOGMA [63] and MITOFY [27], respectively. Intron nomenclature for mitochondrial genes follows Dombrovska and Qiu [64]. All tRNA genes were predicted using tRNAscan-SE v.1.3.1 [65]. Sequences of the annotated organelle genomes were deposited in GenBank (accession numbers KJ485849 and KJ485850). Genome maps were drawn with OGDRAW [66].

Repetitive sequences were identified by performing BlastN v.2.2.28+ comparisons of the *Rhazya* mitochondrial genome against itself with an e-value cutoff of 1e-10 and at least 90% sequence identity. Genome maps were drawn with Circos v.0.64 [67]. Repeat regions involved in recombination were identified by mapping of the corrected PacBio reads. To test for alternative recombinogenic conformations of the mitochondrial genome, polymerase chain reaction (PCR) was carried out using total genomic DNA and primers designed by Primer3 [68] in Geneious R6 (Additional file 1: Table S12).

Open Reading Frames (ORFs) longer than 300 bp in *Rhazya* mitochondrial genome were analyzed using the ORF finder from the National Center for Biotechnology Information (NCBI) [69]. Any ORFs that overlapped with annotated *Rhazya* mitochondrial genes and genes transferred from the plastid were excluded. To search for chimeric ORFs, all ORFs longer than 150 bp were compared with annotated *Rhazya* mitochondrial genes using BlastN with an e-value cutoff of 1e-3, minimum length of 30 bp (as described in Mower et al. [22]) and at least 90% sequence identity. Transmembrane helices in detected ORFs were predicted using TMHMM Server v.2.0 [70].

Plastid-like sequences transferred to the mitochondrial genome were identified by performing BlastN searches of *Rhazya* plastid genome against the mitochondrial genome in Geneious R6 with an e-value cutoff of 1e-10, at least 80% sequence identity and minimum length of 50 bp. Mitochondrial-encoded genes (CDS) were used as BlastN queries against the *Rhazya* plastid genome to search for gene sequences transferred into the plastid genome. To identify putative transposable elements (TEs), the mitochondrial genome was searched against CENSOR web server [71] with default parameters and ‘green plants’ as a reference sequence source. In addition to the *Rhazya* mitochondrial genome, seven other asterid mitochondrial genomes (*Asclepias syriaca*; NC_022796, *Boea hygrometrica*; NC_002511, *Daucus carota* subsp. *sativus*; NC_017855, *Helianthus annuus*; NC_023337, *Mimulus guttatus*; NC_018041, *Nicotiana tabacum*; NC_006581, and *Vaccinium macrocarpon*; NC_023338) were examined for repetitive sequences, plastid-like sequences and TEs. To search for plastid-like sequences in mitochondrial genomes, their plastid counterparts (*Asclepias syriaca*; NC_022432, *Boea hygrometrica*; NC_016468, *Daucus carota* subsp. *sativus*; NC_008325, *Helianthus annuus*; NC_007977, *Jasminum nudiflorum* [for *Mimulus guttatus*]; NC_008407, *Nicotiana tabacum*; NC_001879, and *Vaccinium macrocarpon*; NC_019616) were used.

RNA editing sites were predicted using PREP-Mt [72] with a cutoff value of 0.5 and PREPACT v.2.12.2 [73] using default settings.

### RNA isolation, transcriptome sequencing, and RT-PCR

Total RNA isolation, library construction and Illumina sequencing were performed according to Zhang et al. [74]. Duplex specific nuclease normalization (Evrogen, Moscow, Russia) of the RNA samples, Illumina RNAseq library construction and sequencing were carried out at the GSAF. Raw read output from *R. stricta* RNAseq was deposited in the small read archive (SRA) at the NCBI (accession number SRR1151604).

To confirm a putative transfer of the nuclear gene (R)-mandelonitrile lyase to the mitochondrion, reverse transcription was performed with gene-specific primers using ImProm-II™ Reverse Transcriptase (Promega, USA). PCR amplification was done with primer pairs specific to the ORFs (Additional file 1: Table S12), the products were treated with ExoSAP-IT (New England Biolabs, Ipswich, MA) and Sanger sequenced at the University of Texas Institute of Cellular and Molecular Biology core facility.

### Identification of genes transferred to the nuclear genome

*Rhazya de novo* transcriptome assembly was performed using Trinity [75], released on 2013-02-25 with the script used in Zhang et al. [74] at TACC. The transferred genes were sought using BlastN (e-value cutoff of 1e-10) of the 38 *Rhazya* mitochondrial-encoded genes and the pseudogene of *sdh3* against the transcriptome contigs. TargetP v.1.1 [76] and Predotar v.1.03 [77] were used to predict mitochondrial targeting presequences (mTP). Putative ORFs were searched using Phytozome v.9.1 [78] with BLASTX and ‘asterid’ as a reference sequence source to identify plant gene families. The NCBI Conserved Domain Database (CDD) was used for functional domain annotation [79]. Nucleotide and amino acid sequences of nuclear and mitochondrial genes were aligned with MUSCLE [80] in Geneious R6.

### Phylogenetic analysis of *rps14* and *sdh3* genes

Phylogenetic analyses were performed on data sets of two genes transferred to the nucleus, *rps14* and *sdh3*. Each data set included mitochondrial and nuclear copies of both genes (Additional file 1: Table S13), and the data sets were aligned with MUSCLE [80] in Geneious R6. Maximum likelihood phylogenetic trees were constructed using RAxML v.7.2.8 [81] with the ‘GTRGAMMA’ evolutionary model under the rapid bootstrap algorithm with 1000 replicates at TACC.

## Availability of supporting data

All supporting data is included as additional files. Complete mitochondrial and plastid genome sequences have been submitted to GenBank (accession numbers KJ485849 and KJ485850) and raw sequencing reads from RNAseq were deposited in the small read archive (SRA) at the NCBI (accession number SRR1151604).

## Electronic supplementary material

Additional file 1:Table S1: The gene content of the *Rhazya stricta* mitochondrial genome. **Table S2**. Predicted repeat pairs in the *Rhazya* mitochondrial genome. **Table S3**. Putative transposable elements (TEs) in the *Rhazya* mitochondrial genome. **Table S4**. Fourteen putative transposable elements (TEs) located in the genic regions. **Table S5**. Blast results of ORFs (>300 bp) in the *Rhazya* mitochondrial genome. **Table S6**. Potential chimeric ORFs. **Table S7**. Blast result of plastid-derived DNA segments in the mitochondrial genome of *Rhazya stricta*. **Table S8**. Predicted RNA editing in 38 protein-coding genes for the *Rhazya* mitochondrial genome. **Table S9**. RNA editing validation of 11 genes using transcriptome data. **Table S10**. Genome size, GC content, repetitive DNA, plastid-like DNA and transposable elements in eight asterid mitochondrial genomes. **Table S11**. *Rhazya* rRNA and tRNA content compared to other asterids and two representative angiosperms. **Table S12**. Primers used for testing alternative recombinogenic conformations and for confirming ORFs that represented the (R)-mandelonitrile lyase gene in the *Rhazya* mitochondrial genome. **Table S13**. Information on the phylogenetic analyses and alignment of the *rps14* and *sdh3* genes. (DOCX 164 KB)

Additional file 2: Figure S1: Seven families of repeats (R1-R7) of *Rhazya* mitochondrial genome involved in recombination. **Figure S2**. PCR strategy for identifying intramolecular recombination across mitochondrial repeats. **Figure S3**. The two mitochondrial ORFs representing the nuclear (R)-mandelonitrile lyase sequence [82]. **Figure S4**. Potential chimeric ORFs. **Figure S5**. Conserved mitochondrial gene blocks among eight asterids. (PDF 4 MB)
