# Supplementary material for: Complete sequences of organelle genomes from the medicinal plant Rhazya stricta (Apocynaceae) and contrasting patterns of mitochondrial genome evolution across asterids
Source: BMC Genomics. 2014 May 28;15(1):405. doi: 10.1186/1471-2164-15-405 (PMC4045975; doi:10.1186/1471-2164-15-405)
Supplement: Supplementary file 1 — Additional file 1:Table S1: The gene content of the Rhazya stricta mitochondrial genome. Table S2. Predicted repeat pairs in the Rhazya mitochondrial genome. Table S3. Putative transposable elements (TEs) in the Rhazya mitochondrial genome. Table S4. Fourteen putative transposable elements (TEs) located in the genic regions. Table S5. Blast results of ORFs (>300 bp) in the Rhazya mitochondrial genome. Table S6. Potential chimeric ORFs. Table S7. Blast result of plastid-derived DNA segments in the mitochondrial genome of Rhazya stricta. Table S8. Predicted RNA editing in 38 protein-coding genes for the Rhazya mitochondrial genome. Table S9. RNA editing validation of 11 genes using transcriptome data. Table S10. Genome size, GC content, repetitive DNA, plastid-like DNA and transposable elements in eight asterid mitochondrial genomes. Table S11. Rhazya rRNA and tRNA content compared to other asterids and two representative angiosperms. Table S12. Primers used for testing alternative recombinogenic conformations and for confirming ORFs that represented the (R)-mandelonitrile lyase gene in the Rhazya mitochondrial genome. Table S13. Information on the phylogenetic analyses and alignment of the rps14 and sdh3 genes. (DOCX 164 KB) [file 12864_2014_6093_MOESM1_ESM.docx]

**Table S1**. Gene content of Rhazya stricta mitochondrial genome.

| Genes of Mitochondrial Origin | |  |
| --- | --- | --- |
| Protein genes | NADH Dehydrogenase Subunits (complex I) | *nad1, nad2, nad3, nad4, nad4L, nad5, nad6, nad7, nad9* (x2) |
|  | Succinate Dehydrogenase Subunits (complex II) | *ψsdh3, sdh4* |
|  | Cytochrome bc1 Complex Subunits (complex III) | *cob* |
|  | Cytochrome c Oxidase Subunits (complex IV) | *cox1, cox2, cox3* |
|  | ATP Synthase Subunits (complex V) | *atp1, atp4, atp6, atp8, atp9* |
|  | Cytochrome c Maturation Proteins | *ccmB, ccmC, ccmFc* (x2), *ccmFn* |
|  | Ribosomal Proteins | *rpl2, rpl5* (x2), *rpl10, rpl16* |
|  |  | *rps1* (x2), *rps3, rps4, rps7, rps10, rps12, rps13*( x2), *rps14* (x2), *rps19* |
|  | Putative Protein Transporter | *mttB* |
|  | Maturases | *matR* |
| RNA genes | Ribosomal RNAs | *rrn26, rrn18, rrn5* |
|  | Transfer RNAs | *trnC-GCA, trnE-UUC, trnF-GAA, trnG-GCC, trnI-CAU, trnK-UUU,* |
|  |  | *trnfM-CAU, trnP-UGG, trnQ-UUG, trnS-GCU, trnS-UGA, trnY-GUA* |
| Genes of Plastid Origin | |  |
| Intact genes | Protein coding genes | *ndhH, atpH, psaA* (x2), *psaB* (x2) |
|  | Transfer RNAs | *trnD-GUC, trnH-GUG, trnI-CAU* (x2), *trnN-GUU, trnS-GGA, trnV-GAC* |
|  |  | *trnW-CCA* |
| Pesudogenes |  | *ψatpI, ψpsbJ, ψpsbL, ψpsbF, ψtrnI-CAU, ψtrnP-UGG* |
| Fragments |  | see Table S7 |

**Table S2.** Predicted repeat pairs in Rhazya mitochondrial genome.

| Identity | Alignment length | Number of mismatches | Number of gap opens | Start1 | End1 | Start2 | End2 | E-value | Bit score |
| --- | --- | --- | --- | --- | --- | --- | --- | --- | --- |
| 99.95 | 36624 | 15 | 1 | 228228 | 264848 | 471125 | 507748 | 0 | 6.60E+04 |
| 100 | 32631 | 0 | 0 | 438029 | 470659 | 548608 | 515978 | 0 | 5.89E+04 |
| 97.83 | 1472 | 29 | 1 | 298091 | 299559 | 470098 | 471569 | 0 | 2509 |
| 99.82 | 562 | 1 | 0 | 298091 | 298652 | 516539 | 515978 | 0 | 1009 |
| 92.61 | 352 | 23 | 1 | 81572 | 81923 | 432399 | 432051 | 7.00E-145 | 517 |
| 92.35 | 183 | 8 | 2 | 298456 | 298632 | 450809 | 450627 | 7.00E-69 | 264 |
| 92.35 | 183 | 8 | 2 | 298456 | 298632 | 535828 | 536010 | 7.00E-69 | 264 |
| 92.35 | 183 | 8 | 2 | 450627 | 450809 | 470639 | 470463 | 7.00E-69 | 264 |
| 92.35 | 183 | 8 | 2 | 450627 | 450809 | 515998 | 516174 | 7.00E-69 | 264 |
| 92.35 | 183 | 8 | 2 | 470463 | 470639 | 535828 | 536010 | 7.00E-69 | 264 |
| 92.35 | 183 | 8 | 2 | 515998 | 516174 | 536010 | 535828 | 7.00E-69 | 264 |
| 96.4 | 139 | 4 | 1 | 210628 | 210766 | 507700 | 507837 | 6.00E-57 | 224 |
| 99.25 | 134 | 1 | 0 | 248168 | 248301 | 402656 | 402789 | 9.00E-61 | 237 |
| 99.25 | 134 | 1 | 0 | 402656 | 402789 | 491068 | 491201 | 9.00E-61 | 237 |
| 100 | 129 | 0 | 0 | 257498 | 257626 | 316042 | 315914 | 1.00E-59 | 233 |
| 100 | 129 | 0 | 0 | 315914 | 316042 | 500526 | 500398 | 1.00E-59 | 233 |
| 92.97 | 128 | 6 | 2 | 306750 | 306877 | 514055 | 513931 | 5.00E-45 | 185 |
| 99.21 | 126 | 1 | 0 | 110391 | 110516 | 325537 | 325662 | 2.00E-56 | 223 |
| 100 | 116 | 0 | 0 | 184714 | 184829 | 299821 | 299706 | 1.00E-52 | 210 |
| 90.62 | 96 | 5 | 2 | 169397 | 169488 | 419708 | 419613 | 1.00E-27 | 127 |
| 94.68 | 94 | 5 | 0 | 216519 | 216612 | 308293 | 308386 | 1.00E-33 | 147 |
| 100 | 93 | 0 | 0 | 262922 | 263014 | 407449 | 407357 | 4.00E-40 | 168 |
| 100 | 93 | 0 | 0 | 407357 | 407449 | 505914 | 505822 | 4.00E-40 | 168 |
| 93.33 | 90 | 6 | 0 | 140605 | 140694 | 169386 | 169475 | 2.00E-30 | 136 |
| 93.1 | 87 | 5 | 1 | 206487 | 206572 | 371194 | 371108 | 1.00E-27 | 127 |
| 95.06 | 81 | 2 | 1 | 140616 | 140694 | 419708 | 419628 | 4.00E-27 | 125 |
| 92.59 | 81 | 5 | 1 | 28755 | 28834 | 95793 | 95873 | 2.00E-24 | 116 |
| 92.59 | 81 | 4 | 2 | 266220 | 266299 | 513979 | 514058 | 3.00E-23 | 113 |
| 100 | 79 | 0 | 0 | 184874 | 184952 | 306824 | 306746 | 2.00E-32 | 143 |
| 98.61 | 72 | 1 | 0 | 247485 | 247556 | 248251 | 248322 | 4.00E-27 | 125 |
| 98.61 | 72 | 1 | 0 | 247485 | 247556 | 491151 | 491222 | 4.00E-27 | 125 |
| 98.61 | 72 | 1 | 0 | 248251 | 248322 | 490385 | 490456 | 4.00E-27 | 125 |
| 98.61 | 72 | 1 | 0 | 490385 | 490456 | 491151 | 491222 | 4.00E-27 | 125 |
| 100 | 71 | 0 | 0 | 207440 | 207510 | 278406 | 278476 | 4.00E-28 | 129 |
| 97.18 | 71 | 2 | 0 | 421379 | 421449 | 468429 | 468499 | 2.00E-25 | 120 |
| 97.18 | 71 | 2 | 0 | 421379 | 421449 | 518208 | 518138 | 2.00E-25 | 120 |
| 90.91 | 66 | 6 | 0 | 187243 | 187308 | 252413 | 252348 | 3.00E-17 | 93.3 |
| 90.91 | 66 | 6 | 0 | 187243 | 187308 | 495313 | 495248 | 3.00E-17 | 93.3 |
| 98.46 | 65 | 0 | 1 | 188644 | 188708 | 317193 | 317256 | 3.00E-22 | 109 |
| 93.75 | 64 | 4 | 0 | 248119 | 248182 | 455702 | 455639 | 6.00E-19 | 98.7 |
| 93.75 | 64 | 4 | 0 | 248119 | 248182 | 530935 | 530998 | 6.00E-19 | 98.7 |
| 93.75 | 64 | 4 | 0 | 455639 | 455702 | 491082 | 491019 | 6.00E-19 | 98.7 |
| 93.75 | 64 | 4 | 0 | 491019 | 491082 | 530935 | 530998 | 6.00E-19 | 98.7 |
| 96.83 | 63 | 2 | 0 | 78183 | 78245 | 386167 | 386105 | 4.00E-21 | 105 |
| 98.39 | 62 | 1 | 0 | 139252 | 139313 | 265853 | 265792 | 1.00E-21 | 107 |
| 95.16 | 62 | 2 | 1 | 28197 | 28257 | 281519 | 281458 | 7.00E-18 | 95.1 |
| 91.67 | 60 | 5 | 0 | 95809 | 95868 | 192551 | 192610 | 4.00E-15 | 86 |
| 93.22 | 59 | 4 | 0 | 140259 | 140317 | 248246 | 248188 | 3.00E-16 | 89.7 |
| 93.22 | 59 | 4 | 0 | 140259 | 140317 | 491146 | 491088 | 3.00E-16 | 89.7 |
| 91.53 | 59 | 5 | 0 | 140259 | 140317 | 402734 | 402676 | 1.00E-14 | 84.2 |
| 91.53 | 59 | 4 | 1 | 253610 | 253668 | 290774 | 290831 | 2.00E-13 | 80.6 |
| 91.53 | 59 | 4 | 1 | 290774 | 290831 | 496510 | 496568 | 2.00E-13 | 80.6 |
| 98.25 | 57 | 1 | 0 | 256729 | 256785 | 290839 | 290895 | 6.00E-19 | 98.7 |
| 98.25 | 57 | 1 | 0 | 290839 | 290895 | 499629 | 499685 | 6.00E-19 | 98.7 |
| 100 | 56 | 0 | 0 | 267403 | 267458 | 281410 | 281465 | 5.00E-20 | 102 |
| 91.07 | 56 | 4 | 1 | 63164 | 63218 | 338116 | 338061 | 7.00E-12 | 75.2 |
| 90.91 | 55 | 2 | 1 | 51677 | 51728 | 279698 | 279752 | 7.00E-12 | 75.2 |
| 90.74 | 54 | 4 | 1 | 281116 | 281169 | 413371 | 413423 | 8.00E-11 | 71.6 |
| 96.23 | 53 | 1 | 1 | 28799 | 28850 | 318179 | 318231 | 1.00E-14 | 84.2 |
| 98.08 | 52 | 1 | 0 | 76556 | 76607 | 169814 | 169865 | 3.00E-16 | 89.7 |
| 100 | 51 | 0 | 0 | 247485 | 247535 | 402739 | 402789 | 3.00E-17 | 93.3 |
| 100 | 51 | 0 | 0 | 402739 | 402789 | 490385 | 490435 | 3.00E-17 | 93.3 |
| 96.08 | 51 | 2 | 0 | 209711 | 209761 | 389585 | 389635 | 1.00E-14 | 84.2 |
| 96.08 | 51 | 2 | 0 | 222298 | 222348 | 256535 | 256585 | 1.00E-14 | 84.2 |
| 96.08 | 51 | 2 | 0 | 222298 | 222348 | 499435 | 499485 | 1.00E-14 | 84.2 |
| 94.12 | 51 | 1 | 2 | 182395 | 182443 | 322152 | 322202 | 8.00E-11 | 71.6 |
| 96 | 50 | 2 | 0 | 298642 | 298691 | 366669 | 366620 | 5.00E-14 | 82.4 |
| 96 | 50 | 2 | 0 | 366620 | 366669 | 470698 | 470649 | 5.00E-14 | 82.4 |
| 100 | 49 | 0 | 0 | 210628 | 210676 | 264800 | 264848 | 3.00E-16 | 89.7 |
| 97.96 | 49 | 1 | 0 | 93819 | 93867 | 255223 | 255175 | 1.00E-14 | 84.2 |
| 97.96 | 49 | 1 | 0 | 93819 | 93867 | 498123 | 498075 | 1.00E-14 | 84.2 |
| 95.92 | 49 | 2 | 0 | 206350 | 206398 | 250586 | 250538 | 2.00E-13 | 80.6 |
| 95.92 | 49 | 2 | 0 | 206350 | 206398 | 493486 | 493438 | 2.00E-13 | 80.6 |
| 95.83 | 48 | 2 | 0 | 278494 | 278541 | 318231 | 318184 | 6.00E-13 | 78.8 |
| 100 | 47 | 0 | 0 | 115798 | 115844 | 183588 | 183542 | 4.00E-15 | 86 |
| 95.74 | 47 | 2 | 0 | 110898 | 110944 | 267104 | 267058 | 2.00E-12 | 77 |
| 93.62 | 47 | 3 | 0 | 209166 | 209212 | 333475 | 333521 | 8.00E-11 | 71.6 |
| 100 | 46 | 0 | 0 | 206428 | 206473 | 372423 | 372468 | 1.00E-14 | 84.2 |
| 97.78 | 45 | 1 | 0 | 73334 | 73378 | 257467 | 257511 | 2.00E-12 | 77 |
| 97.78 | 45 | 1 | 0 | 73334 | 73378 | 500367 | 500411 | 2.00E-12 | 77 |
| 97.78 | 45 | 1 | 0 | 183542 | 183586 | 249195 | 249151 | 2.00E-12 | 77 |
| 97.78 | 45 | 1 | 0 | 183542 | 183586 | 492095 | 492051 | 2.00E-12 | 77 |
| 100 | 44 | 0 | 0 | 153462 | 153505 | 153530 | 153573 | 2.00E-13 | 80.6 |
| 97.73 | 44 | 1 | 0 | 212836 | 212879 | 223981 | 223938 | 7.00E-12 | 75.2 |
| 97.67 | 43 | 1 | 0 | 112570 | 112612 | 201332 | 201290 | 2.00E-11 | 73.4 |
| 100 | 42 | 0 | 0 | 138678 | 138719 | 141802 | 141761 | 2.00E-12 | 77 |
| 100 | 39 | 0 | 0 | 318274 | 318312 | 364278 | 364240 | 8.00E-11 | 71.6 |

**Table S3.** Putative transposable elements (TEs) in Rhazya mitochondrial genome.

| From | To | Name | From | To | Class | Dir | Sim | Pos/Mm:Ts | Score |
| --- | --- | --- | --- | --- | --- | --- | --- | --- | --- |
| 9229 | 9441 | Gypsy-79_PTr-LTR | 1149 | 1339 | LTR/Gypsy | c | 0.7181 | 2.625 | 287 |
| 15430 | 15587 | Copia-48_GM-I | 2150 | 2307 | LTR/Copia | c | 0.6981 | 2.4211 | 389 |
| 17482 | 17561 | Gypsy-34_VV-I | 4491 | 4570 | LTR/Gypsy | c | 0.6875 | 1.7857 | 260 |
| 18065 | 18187 | Gypsy-34_VV-I | 3470 | 3596 | LTR/Gypsy | c | 0.7107 | 1.8824 | 352 |
| 18545 | 18811 | Gypsy-34_VV-I | 2772 | 3054 | LTR/Gypsy | c | 0.6704 | 1.5769 | 623 |
| 26047 | 26126 | RETROSAT5_LTR | 1449 | 1526 | LTR/Gypsy | d | 0.7342 | 1.8 | 236 |
| 28847 | 28894 | RLG_scMaximus_1_4-I | 2080 | 2130 | LTR/Copia | d | 0.82 | 3.5 | 222 |
| 41999 | 42169 | Copia-44_PX-I | 3158 | 3324 | LTR/Copia | d | 0.7143 | 2 | 383 |
| 43983 | 44023 | EnSpm-3_ALy | 7531 | 7576 | DNA/EnSpm/CACTA | c | 0.8837 | 1.5 | 237 |
| 47302 | 47354 | Gypsy-64_GM-LTR | 202 | 258 | LTR/Gypsy | c | 0.7963 | 1.8 | 205 |
| 53051 | 53134 | Copia-76_ALY-I | 192 | 267 | LTR/Copia | d | 0.7595 | 2 | 247 |
| 57542 | 57582 | VANDAL18 | 5610 | 5654 | DNA/MuDR | d | 0.8605 | 1 | 253 |
| 60847 | 60942 | Gypsy-21_VV-I | 1722 | 1812 | LTR/Gypsy | d | 0.7021 | 1.4375 | 233 |
| 62575 | 62679 | RLG_scDEL_5_1-I | 3880 | 3986 | LTR/Gypsy | c | 0.6852 | 1.6111 | 270 |
| 72865 | 72926 | Copia-10_EPa-I | 1674 | 1735 | LTR/Copia | d | 0.7581 | 1.8571 | 237 |
| 77175 | 77242 | Gypsy-13_Mad-I | 6896 | 6964 | LTR/Gypsy | d | 0.75 | 2.1429 | 257 |
| 77426 | 77489 | Copia-39_BRa-I | 33 | 103 | LTR/Copia | d | 0.7647 | 1.5 | 252 |
| 81488 | 81571 | OGRE-I_PS | 7177 | 7264 | LTR/Gypsy | c | 0.7674 | 2.5714 | 346 |
| 81978 | 82065 | Copia-96_GM-I | 2496 | 2578 | LTR/Copia | c | 0.7176 | 1.6923 | 232 |
| 82630 | 82687 | Copia20-VV_I | 249 | 308 | LTR/Copia | d | 0.8103 | 1.6 | 236 |
| 83079 | 83139 | Copia38-PTR_I | 289 | 344 | LTR/Copia | d | 0.7667 | 1.25 | 226 |
| 87037 | 87110 | EnSpm-38_SBi | 3211 | 3278 | DNA/EnSpm/CACTA | c | 0.7286 | 1.6 | 211 |
| 87857 | 87895 | Gypsy-31_Mad-LTR | 1094 | 1132 | LTR/Gypsy | d | 0.8462 | 3 | 232 |
| 92039 | 92187 | Gypsy2-VV_I | 345 | 498 | LTR/Gypsy | d | 0.7105 | 2.1111 | 429 |
| 95661 | 95799 | Gypsy-133_GM-LTR | 277 | 391 | LTR/Gypsy | d | 0.7417 | 1.7143 | 202 |
| 109665 | 109735 | MuDR-2_ALy | 4743 | 4817 | DNA/MuDR | d | 0.8143 | 1.1667 | 217 |
| 112415 | 112482 | Copia-3_SMo-I | 822 | 886 | LTR/Copia | d | 0.803 | 4.5 | 243 |
| 112545 | 112594 | RLG_scMaximus_1_2-I | 4031 | 4085 | LTR/Copia | d | 0.8431 | 99 | 251 |
| 116074 | 116127 | DNA3-4_Mad | 132 | 187 | DNA/Harbinger | c | 0.8214 | 1.75 | 249 |
| 118645 | 118697 | Gypsy-66_PAb-I | 11509 | 11561 | LTR/Gypsy | d | 0.7925 | 1.125 | 257 |
| 123807 | 123855 | Gypsy-77_GM-I | 4477 | 4524 | LTR/Gypsy | c | 0.7755 | 1.6667 | 226 |
| 125481 | 125585 | LTR-19_Mad | 99 | 197 | LTR/Copia | c | 0.72 | 1.6923 | 209 |
| 130461 | 130503 | Gypsy-10_ALY-I | 3422 | 3464 | LTR/Gypsy | c | 0.7674 | 1.4286 | 221 |
| 139949 | 139980 | Copia-81_ALY-I | 244 | 274 | LTR/Copia | c | 0.9375 | 99 | 217 |
| 144100 | 144188 | Copia-36_FV-I | 1630 | 1723 | LTR/Copia | d | 0.8242 | 1.8571 | 459 |
| 145424 | 145473 | Copia-26_GM-I | 425 | 474 | LTR/Copia | c | 0.7843 | 1.5 | 203 |
| 145924 | 146143 | SHALINE5_MT | 3569 | 3800 | NonLTR/L1 | d | 0.6545 | 1.7568 | 201 |
| 146212 | 146485 | SHALINE9_MT | 43 | 305 | NonLTR/L1 | d | 0.7061 | 1.6471 | 404 |
| 153843 | 154062 | Gypsy-78_PAb-I | 2185 | 2402 | LTR/Gypsy | c | 0.7453 | 1.6667 | 692 |
| 156312 | 156390 | Gypsy-11_Pru-I | 7392 | 7470 | LTR/Gypsy | c | 0.7 | 1.4286 | 222 |
| 156550 | 156612 | Gypsy-75_PAb-I | 7794 | 7856 | LTR/Gypsy | c | 0.746 | 1.6 | 282 |
| 156665 | 156722 | Gypsy-29_VV-I | 6878 | 6938 | LTR/Gypsy | c | 0.7833 | 2.2 | 241 |
| 161791 | 161845 | Copia-68_GM-LTR | 879 | 933 | LTR/Copia | d | 0.7321 | 1.3 | 203 |
| 167741 | 168073 | Copia-18_BD-I | 4451 | 4769 | LTR/Copia | d | 0.7019 | 1.5 | 978 |
| 168490 | 168521 | Gypsy-167_ZM-I | 4202 | 4235 | LTR/Gypsy | c | 0.8788 | 1 | 208 |
| 178628 | 178691 | Gypsy18-VV_I | 870 | 936 | LTR/Gypsy | c | 0.7385 | 1.6667 | 237 |
| 179190 | 179220 | Copia-34-I_VV | 3351 | 3381 | LTR/Copia | c | 0.9355 | 1 | 263 |
| 179872 | 180121 | SHALINE5_MT | 4834 | 5095 | NonLTR/L1 | c | 0.7167 | 1.8065 | 426 |
| 181288 | 181317 | Gypsy13-PTR_I | 7511 | 7540 | LTR/Gypsy | d | 0.9 | 1.5 | 224 |
| 181661 | 181724 | Gypsy-14_SB-I | 1988 | 2053 | LTR/Gypsy | d | 0.791 | 4.5 | 226 |
| 189272 | 189324 | MuDR-5_ZM | 4564 | 4617 | DNA/MuDR | d | 0.8148 | 2.25 | 241 |
| 190104 | 190158 | TE7-1_FV | 127 | 178 | Interspersed_Repeat | d | 0.7778 | 2 | 210 |
| 197483 | 197545 | Helitron-1_ALy | 10850 | 10919 | DNA/Helitron | c | 0.7727 | 2.4 | 228 |
| 199481 | 199627 | Gypsy-18_ST-I | 2624 | 2778 | LTR/Gypsy | d | 0.6986 | 1.8571 | 343 |
| 206927 | 206977 | ATCOPIA53_I | 56 | 99 | LTR/Copia | c | 0.8723 | 1.5 | 238 |
| 209308 | 209368 | GYPSO_I | 6794 | 6848 | LTR/Gypsy | c | 0.7797 | 1.2857 | 231 |
| 209993 | 210282 | Copia-41_FV-I | 3934 | 4234 | LTR/Copia | d | 0.7143 | 2.0571 | 774 |
| 210548 | 210590 | Gypsy-10_BRa-LTR | 47 | 90 | LTR/Gypsy | c | 0.8182 | 1.4 | 217 |
| 220455 | 220518 | DNA-4_FV | 337 | 400 | DNA | d | 0.7656 | 2.4 | 222 |
| 224692 | 224768 | EnSpm-1_STu | 12017 | 12086 | DNA/EnSpm/CACTA | c | 0.8889 | 3 | 463 |
| 225898 | 225939 | TAM2_AM | 3112 | 3153 | Interspersed_Repeat | c | 0.9048 | 99 | 268 |
| 227576 | 227619 | Helitron-1_SBi | 5932 | 5973 | DNA/Helitron | d | 0.814 | 1.4 | 231 |
| 227976 | 228117 | SHALINE4_MT | 1976 | 2115 | NonLTR/L1 | d | 0.6761 | 2.1111 | 214 |
| 230804 | 230844 | Copia-69_BRa-I | 3715 | 3755 | LTR/Copia | c | 0.9024 | 4 | 302 |
| 231180 | 231394 | Copia-67_ST-I | 3147 | 3365 | LTR/Copia | c | 0.6759 | 1.6765 | 285 |
| 233919 | 234230 | Monkey_MA | 2687 | 3011 | LTR/Gypsy | d | 0.6935 | 1.7143 | 774 |
| 234887 | 234940 | hAT-8_SBi | 1252 | 1307 | DNA/hAT | c | 0.8 | 2.5 | 267 |
| 236555 | 236592 | ATLANTYS3I | 4853 | 4890 | LTR/Gypsy | c | 0.9474 | 1 | 314 |
| 238075 | 238218 | Gypsy9-VV_I | 2133 | 2276 | LTR/Gypsy | c | 0.7083 | 1.68 | 582 |
| 238219 | 238264 | Gypsy-5_ST-I | 1388 | 1433 | LTR/Gypsy | c | 0.7609 | 1.375 | 230 |
| 238879 | 239081 | Gypsy-30_SB-I | 3555 | 3757 | LTR/Gypsy | c | 0.665 | 1.8485 | 424 |
| 240268 | 240310 | Copia-93_ST-I | 2708 | 2751 | LTR/Copia | d | 0.8409 | 1.25 | 227 |
| 247294 | 247363 | EnSpm-8_ALy | 958 | 1025 | DNA/EnSpm/CACTA | d | 0.7571 | 1.4444 | 212 |
| 249488 | 249521 | Copia-62_GM-I | 252 | 285 | LTR/Copia | d | 0.8235 | 1 | 228 |
| 252816 | 252890 | MuDR-11_ALy | 2456 | 2534 | DNA/MuDR | d | 0.75 | 2.2857 | 236 |
| 258429 | 258474 | Copia-29-I_VV | 3586 | 3633 | LTR/Copia | d | 0.7872 | 1.5 | 229 |
| 260629 | 260659 | EnSpm-2_ALy | 469 | 499 | DNA/EnSpm/CACTA | c | 0.871 | 1 | 206 |
| 260982 | 261021 | Copia-88_GM-I | 2643 | 2682 | LTR/Copia | c | 0.8 | 1.6 | 225 |
| 264928 | 264977 | MuDR-4_FV | 1166 | 1211 | DNA/MuDR | d | 0.8542 | 2 | 218 |
| 273385 | 273506 | Copia-65_Mad-I | 5989 | 6105 | LTR/Copia | d | 0.775 | 2 | 476 |
| 275313 | 275344 | Ogre-MT3_LTR | 178 | 209 | LTR/Gypsy | c | 0.9062 | 3 | 204 |
| 275448 | 275497 | Copia-39_Mad-I | 3539 | 3593 | LTR/Copia | c | 0.7885 | 1.5 | 206 |
| 278002 | 278084 | Copia-141_SB-I | 994 | 1073 | LTR/Copia | c | 0.7922 | 2.1667 | 294 |
| 278755 | 278848 | EnSpm-1_STu | 12141 | 12233 | DNA/EnSpm/CACTA | c | 0.8511 | 1.3 | 586 |
| 281474 | 281528 | Copia-85_Mad-I | 1016 | 1069 | LTR/Copia | c | 0.7857 | 1.5 | 251 |
| 288191 | 288232 | Caulimovirus-1_VVi | 1115 | 1156 | IntegratedVirus/Caulimovirus | d | 0.8095 | 2.6667 | 205 |
| 300707 | 300774 | VIHAT3 | 479 | 544 | DNA/hAT | d | 0.7059 | 1.5 | 200 |
| 300825 | 300879 | L1-15_ALy | 1611 | 1669 | NonLTR/L1 | c | 0.75 | 1.625 | 227 |
| 303009 | 303856 | Copia-4_PD-I | 2975 | 3809 | LTR/Copia | c | 0.8242 | 1.7703 | 4429 |
| 306705 | 306746 | ATCOPIA11I | 3528 | 3569 | LTR/Copia | d | 0.881 | 1.6667 | 278 |
| 310578 | 310615 | RAM9B_LTR | 2239 | 2277 | LTR/Gypsy | c | 0.8205 | 1 | 226 |
| 311651 | 311696 | Gypsy-28_ALY-I | 2799 | 2847 | LTR/Gypsy | c | 0.7872 | 1.1429 | 213 |
| 312332 | 312395 | Ogre-SD1_I | 5272 | 5336 | LTR/Gypsy | d | 0.7385 | 1.6667 | 212 |
| 316195 | 316273 | Mariner-1_GM | 3272 | 3350 | DNA/Mariner | d | 0.9367 | 2.5 | 626 |
| 316707 | 316792 | CLAUDIA1_TM | 2317 | 2395 | LTR/Copia | d | 0.7439 | 1.25 | 238 |
| 316973 | 317018 | Helitron-2_STu | 4329 | 4374 | DNA/Helitron | d | 0.9348 | 1.5 | 373 |
| 322996 | 323081 | Copia15-ZM_I | 6284 | 6361 | LTR/Copia | d | 0.679 | 1.2105 | 233 |
| 327170 | 327283 | Copia-36_VV-I | 2931 | 3046 | LTR/Copia | c | 0.7018 | 1.8824 | 334 |
| 340866 | 340927 | MEGY-I_MT | 1265 | 1325 | LTR/Gypsy | c | 0.7258 | 1.7778 | 216 |
| 342683 | 342738 | HARB-1N1_STu | 722 | 780 | DNA/Harbinger | c | 0.7719 | 1.375 | 205 |
| 344400 | 344452 | Gypsy-20_SMo-I | 6061 | 6113 | LTR/Gypsy | d | 0.7736 | 2 | 247 |
| 351983 | 352031 | EnSpm-1_STu | 11744 | 11791 | DNA/EnSpm/CACTA | d | 0.7755 | 2 | 230 |
| 352147 | 352205 | MuDR-N1_ZM | 937 | 995 | DNA/MuDR | c | 0.7627 | 1.2727 | 314 |
| 353435 | 353487 | MuDR-12_GM | 9022 | 9074 | DNA/MuDR | d | 0.717 | 1.6667 | 223 |
| 354464 | 354523 | Helitron-2_PTr | 1742 | 1803 | DNA/Helitron | c | 0.7377 | 1.1818 | 208 |
| 356210 | 356277 | L1-1_GM | 4677 | 4738 | NonLTR/L1 | d | 0.7969 | 1.5 | 266 |
| 357885 | 357933 | Gypsy-73_GM-I | 1882 | 1932 | LTR/Gypsy | c | 0.7843 | 1.5 | 241 |
| 359060 | 359121 | Helitron-2B_ALy | 8887 | 8943 | DNA/Helitron | d | 0.8103 | 2.6667 | 227 |
| 359751 | 359842 | Copia-39_BD-I | 3016 | 3111 | LTR/Copia | d | 0.7174 | 1.5 | 230 |
| 360059 | 360150 | VANDAL5A | 602 | 680 | DNA/MuDR | d | 0.7349 | 1.4167 | 230 |
| 360892 | 360947 | Helitron-N3_ZM | 12021 | 12074 | DNA/Helitron | c | 0.7818 | 2.2 | 244 |
| 361237 | 361820 | Copia-97_GM-I | 3225 | 3818 | LTR/Copia | d | 0.6839 | 1.9753 | 1173 |
| 367194 | 367357 | POPCOP2_I | 6206 | 6364 | LTR/Copia | d | 0.7222 | 2.1765 | 409 |
| 367559 | 367616 | Copia10-VV_I | 2603 | 2663 | LTR/Copia | c | 0.8197 | 2 | 225 |
| 367998 | 368029 | MuDR-3_VV | 6087 | 6118 | DNA/MuDR | c | 0.9375 | 1 | 238 |
| 372274 | 372369 | EnSpm-N18_SBi | 530 | 619 | DNA/EnSpm/CACTA | d | 0.7416 | 1.4167 | 210 |
| 383225 | 383265 | Sat-1_CPa | 7061 | 7104 | Simple/Sat | c | 0.8372 | 1.25 | 241 |
| 391517 | 391582 | Copia-45_VV-I | 1466 | 1523 | LTR/Copia | c | 0.7833 | 2.2 | 205 |
| 395131 | 395163 | DNA-13_ALy | 490 | 522 | DNA | d | 0.8485 | 1 | 225 |
| 396423 | 396481 | Gypsy9-VV_I | 4549 | 4607 | LTR/Gypsy | c | 0.7458 | 1.875 | 263 |
| 396525 | 396713 | Monkey_MA | 3275 | 3463 | LTR/Gypsy | c | 0.6825 | 1.8929 | 423 |
| 399030 | 399093 | EnSpm-1_TA | 4585 | 4646 | DNA/EnSpm/CACTA | c | 0.7846 | 2 | 239 |
| 399670 | 399721 | Copia-11_FV-I | 4521 | 4572 | LTR/Copia | c | 0.7692 | 1.0909 | 290 |
| 402883 | 402949 | TRUNCATOR | 2323 | 2397 | LTR/Gypsy | c | 0.7606 | 1.625 | 237 |
| 411979 | 412034 | Copia-74_ST-I | 2909 | 2968 | LTR/Copia | d | 0.7719 | 2 | 247 |
| 417780 | 417824 | Copia-94_Mad-I | 3253 | 3299 | LTR/Copia | c | 0.8261 | 2.3333 | 240 |
| 422754 | 422963 | Gypsy-42_Mad-I | 737 | 943 | LTR/Gypsy | d | 0.6667 | 1.7778 | 535 |
| 425346 | 425463 | Copia-31_Pru-I | 3587 | 3700 | LTR/Copia | c | 0.6609 | 1.6364 | 214 |
| 427922 | 428970 | Gypsy-9_PX-I | 2759 | 3790 | LTR/Gypsy | c | 0.6989 | 1.8867 | 2910 |
| 428988 | 429088 | Gypsy-24_FV-I | 1517 | 1617 | LTR/Gypsy | c | 0.6535 | 1.4583 | 330 |
| 443712 | 443743 | L1-13_FV | 175 | 207 | NonLTR/L1 | d | 0.9091 | 2 | 232 |
| 448815 | 448857 | EnSpm-1_HV | 9823 | 9866 | DNA/EnSpm/CACTA | c | 0.75 | 1.25 | 210 |
| 453556 | 453600 | Copia-33_GM-I | 1542 | 1586 | LTR/Copia | d | 0.8478 | 5 | 241 |
| 453948 | 453975 | EnSpm-26_SBi | 4224 | 4251 | DNA/EnSpm/CACTA | c | 0.9286 | 1 | 220 |
| 454384 | 454436 | Gypsy-2_PX-I | 10077 | 10129 | LTR/Gypsy | d | 0.7925 | 1.8333 | 279 |
| 458940 | 458988 | EnSpm-14_OS | 2970 | 3018 | DNA/EnSpm/CACTA | d | 0.7143 | 1.1667 | 234 |
| 463909 | 464091 | Gypsy-3_ST-I | 3232 | 3415 | LTR/Gypsy | c | 0.6684 | 2.2917 | 371 |
| 464245 | 464348 | Copia-15_CP-I | 2191 | 2305 | LTR/Copia | c | 0.7727 | 1.6364 | 398 |
| 465346 | 465478 | Gypsy-46_Mad-I | 1507 | 1638 | LTR/Gypsy | d | 0.609 | 1.5938 | 233 |
| 465686 | 465722 | Caulimovirus-1_BD | 5087 | 5123 | IntegratedVirus/Caulimovirus | d | 0.8378 | 1.5 | 210 |
| 473704 | 473744 | Copia-69_BRa-I | 3715 | 3755 | LTR/Copia | c | 0.9024 | 4 | 302 |
| 474080 | 474294 | Copia-67_ST-I | 3147 | 3365 | LTR/Copia | c | 0.6759 | 1.6765 | 285 |
| 476819 | 477130 | Monkey_MA | 2687 | 3011 | LTR/Gypsy | d | 0.6935 | 1.7143 | 774 |
| 477787 | 477840 | hAT-8_SBi | 1252 | 1307 | DNA/hAT | c | 0.8 | 2.5 | 267 |
| 479455 | 479492 | ATLANTYS3I | 4853 | 4890 | LTR/Gypsy | c | 0.9474 | 1 | 314 |
| 480975 | 481118 | Gypsy9-VV_I | 2133 | 2276 | LTR/Gypsy | c | 0.7083 | 1.68 | 582 |
| 481119 | 481164 | Gypsy-5_ST-I | 1388 | 1433 | LTR/Gypsy | c | 0.7609 | 1.375 | 230 |
| 481779 | 481981 | Gypsy-30_SB-I | 3555 | 3757 | LTR/Gypsy | c | 0.665 | 1.8485 | 424 |
| 483168 | 483210 | Copia-93_ST-I | 2708 | 2751 | LTR/Copia | d | 0.8409 | 1.25 | 227 |
| 490194 | 490263 | EnSpm-8_ALy | 958 | 1025 | DNA/EnSpm/CACTA | d | 0.7571 | 1.4444 | 212 |
| 492388 | 492421 | Copia-62_GM-I | 252 | 285 | LTR/Copia | d | 0.8235 | 1 | 228 |
| 495716 | 495790 | MuDR-11_ALy | 2456 | 2534 | DNA/MuDR | d | 0.75 | 2.2857 | 236 |
| 501329 | 501374 | Copia-29-I_VV | 3586 | 3633 | LTR/Copia | d | 0.7872 | 1.5 | 229 |
| 503529 | 503559 | EnSpm-2_ALy | 469 | 499 | DNA/EnSpm/CACTA | c | 0.871 | 1 | 206 |
| 503882 | 503921 | Copia-88_GM-I | 2643 | 2682 | LTR/Copia | c | 0.8 | 1.6 | 225 |
| 509996 | 510049 | Copia-86_VV-I | 5267 | 5320 | LTR/Copia | d | 0.8269 | 1.5 | 204 |
| 520915 | 520951 | Caulimovirus-1_BD | 5087 | 5123 | IntegratedVirus/Caulimovirus | c | 0.8378 | 1.5 | 210 |
| 521159 | 521291 | Gypsy-46_Mad-I | 1507 | 1638 | LTR/Gypsy | c | 0.609 | 1.5938 | 233 |
| 522289 | 522392 | Copia-15_CP-I | 2191 | 2305 | LTR/Copia | d | 0.7727 | 1.6364 | 398 |
| 522546 | 522728 | Gypsy-3_ST-I | 3232 | 3415 | LTR/Gypsy | d | 0.6684 | 2.2917 | 371 |
| 527649 | 527697 | EnSpm-14_OS | 2970 | 3018 | DNA/EnSpm/CACTA | c | 0.7143 | 1.1667 | 234 |
| 532201 | 532253 | Gypsy-2_PX-I | 10077 | 10129 | LTR/Gypsy | c | 0.7925 | 1.8333 | 279 |
| 532662 | 532689 | EnSpm-26_SBi | 4224 | 4251 | DNA/EnSpm/CACTA | d | 0.9286 | 1 | 220 |
| 533037 | 533081 | Copia-33_GM-I | 1542 | 1586 | LTR/Copia | c | 0.8478 | 5 | 241 |
| 537780 | 537822 | EnSpm-1_HV | 9823 | 9866 | DNA/EnSpm/CACTA | d | 0.75 | 1.25 | 210 |
| 542894 | 542925 | L1-13_FV | 175 | 207 | NonLTR/L1 | c | 0.9091 | 2 | 232 |

**From/To;** beginning/ending of positions of fragments on corresponding sequence (1^st^&2^nd^ column -submitted *Rhazya* query sequences, 4^th^&5^th^ column Repbase library sequences). **Name;** locus names of Repbase library sequences. **Class;** class/subclass of repeat as specified in repeat annotation. **Dir;** orientation ('d' for direct, 'c' for complementary) of repeat fragment. **Sim**; value of similarity between 2 aligned fragments. **Pos**; the ratio of positives to alignment length. **Mm:Ts;** a ratio of mismatches to transitions in nucleotide alignment. **Score;** the alignment score obtained from blast. The putative transposable elements inserted in genic regions are in red.

**Table S4.** Fourteen putative transposable elements (TEs) located in the genic regions.

| No. | Genic regions | TE class | *Rhazya* | *Asclepias* | *Nicotiana* | *Mimulus* | *Boea* | *Daucus* | *Helianthus* | *Vaccinium* |
| --- | --- | --- | --- | --- | --- | --- | --- | --- | --- | --- |
| 1 | ***cox2 exon1*** | DNA/EnSpm/CACTA | 94 | 94 | 97 | 92 | **-** | 94 | 94 | 94 |
| 2 | ***cox3*** | DNA/Helitron | 46 | 51 | 51 | 52 | 51 | 51 | 51 | 51 |
| 3 | ***cox3*** | See taxon column | 86  LTR/Copia | **-** | 41  DNA/MuDR | **-** | **-** | **-** | **-** | **-** |
| 4 | ***cox3/sdh4**** | DNA/Mariner | 40/39 | 40/39 | 40/39 | 40/39 | 40/39 | 40/47^a^ | 40/49^a^ | 40/28^a^, 79^b^ |
| 5 | *nad2i709* | LTR/Gypsy | 43 | **-** | 43 | **-** | **-** | **-** | 43 | 43 |
| 6 | *nad4i976* | LTR/Copia | 66 | **-** | **-** | **-** | **-** | **-** | ○ | ○ |
| 7 | ***nad5 exon5*** | LTR/Copia | 54 | 61 | **-** | **-** | **-** | **-** | **-** | **-** |
| 8 | ***nad7i209/exon3*** | LTR/Copia | 38/46 | 38/46 | **-** | **-** | **-** | **-** | **-** | **-** |
| 9 | ***rps19*** | DNA/MuDR | 50 | 50 | 50 | x | x | x | 50^ψ^ | 44 |
| 10 | ***rps4*** | LTR/Copia | 51 | - | **-** | 51 | **-** | - | - | - |
| 11 | *rrn26* | LTR/Copia | 333 | 327 | 333 | 209 | 333 | 333 | 261 | 333 |
| 12 | *rrn26* | LTR/Gypsy | 32 | **-** | **-** | **-** | **-** | **-** | **-** | **-** |
| 13 | *trnK-UUU* | DNA/MuDR | 59 | 59 | 59 | 59 | 59 | 59 | 59 | 59 |
| 14 | *intergenic region*  */trnP-UGG* | DNA/Helitron | 26/18 | 26/18 | 26/27 | 26/18 | 26/18 | 26/18 | 26/18 | 26/18 |

The numbers below species indicate a length (bp) of transposable elements. **cox3* and *sdh4* overlap in the mitochondrial genome. ^a^TE fragments are located in intergenic regions (The *sdh4* of *Daucus* and *Helianthus* are absent in their mitochondrial genomes). ^b^TE fragment is located in *sdh4* gene only (The *cox3* and *sdh4* of *Vaccinium* are separated in the mitochondrial genome). ○; intron absent, x; gene loss, ψ; pseudogene. Bold font indicates protein-coding regions.

**Table S5.** Blast results of ORFs (> 300 bp) in Rhazya mitochondrial genome.

|  | From | To | length | transcripts | nuclear DNA^a^ | Hit start | Hit end | Identity | Note |
| --- | --- | --- | --- | --- | --- | --- | --- | --- | --- |
| ORF834 | 2,468 | 4,972 | 2,505 | ○ | ○ | 619 | 1,980 | 68.4 | *DNA-directed DNA polymerases* |
| ORF581 | 436,363 | 438,108 | 1,746 | ● | ○ | - | - | - | No significant similarity found |
| ORF430 | 71,410 | 72,702 | 1,293 | ● | ● | 3 | 1292 | 73.8 | *(R)-mandelonitrile lyase-like* |
| ORF394 | 588 | 1,772 | 1,185 | ● | ● | 227 | 819 | 66.1 | *DNA-directed RNA polymerase* |
|  |  |  |  |  |  | 877 | 1122 | 69.9 | *DNA-dependent RNA polymerase* |
| ORF324 | 470,646 | 471,620 | 975 | ● | ● | 595 | 860 | 74.3 | hypothetical protein |
| ORF322a | 453,957 | 454,925 | 969 | ● | ○ | 23 | 464 | 89.6 | mitochondrial-like sequence |
| ORF322b | 531,712 | 532,680 | 969 | ● | ○ | 23 | 464 | 89.6 | mitochondrial-like sequence |
| ORF318 | 298,639 | 299,595 | 957 | ● | ● | 508 | 866 | 80.5 | hypothetical protein |
| ORF315 | 114,795 | 115,742 | 948 | ● | ○ | 69 | 889 | 66.7 | mitochondrial-like sequence |
| ORF288 | 331,586 | 332,452 | 867 | ○ | ○ | 1 | 489 | 71.7 | *DNA-dependent RNA polymerase* |
| ORF278 | 116,114 | 116,950 | 837 | ○ | ● | 499 | 678 | 70.0 | mitochondrial-like sequence |
| ORF273 | 112,937 | 113,758 | 822 | ● | ○ | - | - | - | No significant similarity found |
| ORF252 | 113,886 | 114,644 | 759 | ● | ○ | 34 | 758 | 66.5 | mitochondrial-like sequence |
| ORF239 | 109,782 | 110,501 | 720 | ● | ○ | 1 | 539 | 72.3 | mitochondrial-like sequence |
| ORF237a* | 449,100 | 449,813 | 714 | ○ | ○ | 316 | 649 | 78.4 | mitochondrial-like sequence |
| ORF237b* | 536,824 | 537,537 | 714 | ○ | ○ | 316 | 649 | 78.4 | mitochondrial-like sequence |
| ORF233 | 15,315 | 16,016 | 702 | ● | ○ | 170 | 620 | 91.6 | mitochondrial-like sequence |
| ORF229 | 8 | 697 | 690 | ● | ● | - | - | - | No significant similarity found |
| ORF218 | 5,934 | 6,590 | 657 | ● | ○ | - | - | - | No significant similarity found |
| ORF206a | 238,344 | 238,964 | 621 | ○ | ● | - | - | - | No significant similarity found |
| ORF206b | 427,964 | 428,584 | 621 | ● | ○ | 1 | 621 | 96.0 | hypothetical protein |
| ORF206c | 481,244 | 481,864 | 621 | ○ | ● | - | - | - | No significant similarity found |
| ORF185 | 71,226 | 71,783 | 558 | ● | ● | 1 | 549 | 72.5 | *(R)-mandelonitrile lyase-like* |
| ORF178 | 18,241 | 18,777 | 537 | ● | ○ | 1 | 233 | 70.3 | mitochondrial-like sequence |
| ORF173 | 219,903 | 220,424 | 522 | ● | ● | 277 | 520 | 73.0 | mitochondrial-like sequence |
| ORF171a | 228,982 | 229,497 | 516 | ● | ○ | 1 | 516 | 97.1 | hypothetical protein |
| ORF171b | 247,546 | 248,061 | 516 | ● | ● | 47 | 182 | 74.1 | mitochondrial-like sequence |
| ORF171c | 471,882 | 472,397 | 516 | ○ | ○ | 1 | 516 | 94.8 | hypothetical protein |
| ORF171d | 490,446 | 490,961 | 516 | ● | ● | 47 | 182 | 74.1 | mitochondrial-like sequence |
| ORF170a | 230,842 | 231,354 | 513 | ● | ○ | - | - | - | No significant similarity found |
| ORF170b | 473,742 | 474,254 | 513 | ● | ○ | - | - | - | No significant similarity found |
| ORF169 | 337,998 | 338,507 | 510 | ○ | ○ | - | - | - | No significant similarity found |
| ORF164 | 75,721 | 76,215 | 495 | ● | ○ | 1 | 491 | 68.0 | *DNA-dependent RNA polymerase* |
| ORF160 | 134,102 | 134,584 | 483 | ● | ○ | 1 | 483 | 94.1 | hypothetical protein |
| ORF159a | 46,909 | 47,388 | 480 | ○ | ○ | 93 | 144 | 93.8 | mitochondrial-like sequence |
| ORF159b | 228,241 | 228,720 | 480 | ● | ● | 213 | 365 | 81.7 | hypothetical protein |
| ORF155a | 337,984 | 338,451 | 468 | ○ | ○ | - | - | - | No significant similarity found |
| ORF155b | 419,596 | 420,063 | 468 | ● | ○ | 96 | 276 | 96.1 | hypothetical protein |
| ORF153 | 28,368 | 28,829 | 462 | ● | ○ | 287 | 451 | 74.7 | mitochondrial-like sequence |
| ORF147 | 375,754 | 376,197 | 444 | ○ | ○ | 248 | 444 | 86.3 | mitochondrial-like sequence |
| ORF144 | 297,112 | 297,546 | 435 | ● | ● | 1 | 330 | 90.7 | mitochondrial-like sequence |
| ORF143b | 457,726 | 458,157 | 432 | ● | ● | - | - | - | No significant similarity found |
| ORF143a | 528,480 | 528,911 | 432 | ● | ● | - | - | - | No significant similarity found |
| ORF142 | 508,027 | 508,455 | 429 | ● | ○ | 127 | 427 | 83.4 | hypothetical protein |
| ORF141a | 29,764 | 30,189 | 426 | ● | ○ | 134 | 424 | 89.4 | mitochondrial-like sequence |
| ORF141b | 81,516 | 81,941 | 426 | ● | ○ | 15 | 368 | 83.6 | hypothetical protein |
| ORF138 | 304,833 | 305,249 | 417 | ● | ○ | 1 | 417 | 91.6 | hypothetical protein |
| ORF133a | 361,580 | 361,981 | 402 | ● | ○ | 50 | 229 | 71.7 | putative retrotransposon polyprotein |
| ORF133b | 380,171 | 380,572 | 402 | ○ | ○ | - | - | - | No significant similarity found |
| ORF131 | 15,319 | 15,714 | 396 | ● | ○ | 79 | 396 | 92.8 | mitochondrial-like sequence |
| ORF129 | 435,314 | 435,703 | 390 | ○ | ○ | - | - | - | No significant similarity found |
| ORF128a | 78,612 | 78,998 | 387 | ● | ○ | 1 | 109 | 95.4 | mitochondrial-like sequence |
| ORF128b | 434,403 | 434,789 | 387 | ● | ○ | - | - | - | No significant similarity found |
| ORF127 | 91,913 | 92,296 | 384 | ○ | ○ | 1 | 384 | 95.4 | mitochondrial-like sequence |
| ORF126a | 156,493 | 156,873 | 381 | ○ | ○ | 110 | 381 | 97.8 | hypothetical protein |
| ORF126b | 171,824 | 172,204 | 381 | ● | ○ | 1 | 381 | 83.3 | mitochondrial-like sequence |
| ORF125a | 358,983 | 359,360 | 378 | ● | ○ | - | - | - | No significant similarity found |
| ORF125b | 432,033 | 432,410 | 378 | ● | ○ | 1 | 369 | 72.5 | hypothetical protein |
| ORF123a | 351,404 | 351,775 | 372 | ○ | ○ | 1 | 372 | 93.8 | hypothetical protein |
| ORF123b | 395,433 | 395,804 | 372 | ● | ○ | 1 | 372 | 91.7 | mitochondrial-like sequence |
| ORF122a | 225,760 | 226,128 | 369 | ● | ○ | 24 | 368 | 87.6 | mitochondrial-like sequence |
| ORF122b | 307,621 | 307,989 | 369 | ● | ○ | 1 | 369 | 88.5 | mitochondrial-like sequence |
| ORF121 | 340,295 | 340,660 | 366 | ○ | ○ | 191 | 285 | 84.0 | mitochondrial-like sequence |
| ORF120a | 9,929 | 10,291 | 363 | ● | ○ | 245 | 363 | 100.0 | mitochondrial-like sequence |
| ORF120b | 133,501 | 133,863 | 363 | ● | ○ | 1 | 363 | 94.8 | mitochondrial-like sequence |
| ORF119a | 251,582 | 251,941 | 360 | ● | ● | 143 | 290 | 78.1 | mitochondrial-like sequence |
| ORF119b | 494,482 | 494,841 | 360 | ● | ● | 143 | 290 | 78.1 | mitochondrial-like sequence |
| ORF118 | 1,830 | 2,186 | 357 | ● | ● | - | - | - | No significant similarity found |
| ORF117 | 103,202 | 103,555 | 354 | ● | ○ | 11 | 354 | 93.0 | mitochondrial-like sequence |
| ORF115a | 135,019 | 135,366 | 348 | ● | ○ | 1 | 348 | 85.8 | mitochondrial-like sequence |
| ORF115b | 152,253 | 152,600 | 348 | ○ | ○ | - | - | - | No significant similarity found |
| ORF115c | 218,232 | 218,579 | 348 | ● | ● | 1 | 283 | 84.9 | mitochondrial-like sequence |
| ORF114a | 321,664 | 322,008 | 345 | ○ | ○ | - | - | - | No significant similarity found |
| ORF114b | 466,326 | 466,670 | 345 | ○ | ○ | 117 | 289 | 85.7 | mitochondrial-like sequence |
| ORF114c | 519,967 | 520,311 | 345 | ○ | ○ | 117 | 289 | 85.7 | mitochondrial-like sequence |
| ORF113a | 146,585 | 146,926 | 342 | ● | ○ | 1 | 339 | 98.5 | mitochondrial-like sequence |
| ORF113b | 362,725 | 363,066 | 342 | ○ | ○ | 1 | 65 | 95.4 | mitochondrial-like sequence |
| ORF113c | 458,653 | 458,994 | 342 | ● | ○ | 266 | 335 | 95.7 | mitochondrial-like sequence |
| ORF113d | 527,643 | 527,984 | 342 | ● | ○ | 266 | 335 | 95.7 | mitochondrial-like sequence |
| ORF112a | 110,545 | 110,883 | 339 | ● | ● | - | - | - | No significant similarity found |
| ORF112b | 247,344 | 247,682 | 339 | ○ | ● | 197 | 283 | 93.7 | mitochondrial-like sequence |
| ORF112c | 331,157 | 331,495 | 339 | ● | ○ | 16 | 298 | 71.7 | *DNA-dependent RNA polymerase* |
| ORF112d | 490,244 | 490,582 | 339 | ○ | ● | 127 | 198 | 93.1 | mitochondrial-like sequence |
| ORF111a | 136,331 | 136,666 | 336 | ● | ○ | 2 | 336 | 97.0 | mitochondrial-like sequence |
| ORF111b | 209,752 | 210,087 | 336 | ● | ○ | 29 | 336 | 78.4 | hypothetical protein |
| ORF111c | 232,559 | 232,894 | 336 | ○ | ○ | 1 | 290 | 87.2 | mitochondrial-like sequence |
| ORF111d | 288,427 | 288,762 | 336 | ● | ○ | 1 | 336 | 93.3 | mitochondrial-like sequence |
| ORF111e | 303,170 | 303,505 | 336 | ● | ● | 1 | 336 | 78.6 | *RNA-dependent DNA polymerase* |
| ORF111f | 332,458 | 332,793 | 336 | ○ | ○ | - | - | - | No significant similarity found |
| ORF111g | 475,459 | 475,794 | 336 | ○ | ○ | 1 | 290 | 87.2 | mitochondrial-like sequence |
| ORF110a | 17,756 | 18,088 | 333 | ● | ● | - | - | - | No significant similarity found |
| ORF110b | 39,365 | 39,697 | 333 | ○ | ○ | 223 | 333 | 89.2 | mitochondrial-like sequence |
| ORF110c | 202,406 | 202,738 | 333 | ● | ● | 235 | 324 | 82.8 | mitochondrial-like sequence |
| ORF110d | 246,213 | 246,545 | 333 | ● | ● | 1 | 95 | 98.9 | mitochondrial-like sequence |
| ORF110e | 360,158 | 360,490 | 333 | ● | ○ | - | - | - | No significant similarity found |
| ORF110f | 489,113 | 489,445 | 333 | ● | ● | 1 | 95 | 98.9 | mitochondrial-like sequence |
| ORF109a | 238,172 | 238,501 | 330 | ○ | ● | - | - | - | No significant similarity found |
| ORF109b | 418,621 | 418,950 | 330 | ● | ● | 54 | 313 | 85.0 | mitochondrial-like sequence |
| ORF109c | 481,072 | 481,401 | 330 | ○ | ● | - | - | - | No significant similarity found |
| ORF107a | 109,383 | 109,706 | 324 | ○ | ○ | 1 | 94 | 93.7 | mitochondrial-like sequence |
| ORF107b | 276,800 | 277,123 | 324 | ○ | ○ | - | - | - | No significant similarity found |
| ORF107c | 354,717 | 355,040 | 324 | ● | ○ | 1 | 320 | 97.8 | mitochondrial-like sequence |
| ORF107d | 452,446 | 452,769 | 324 | ● | ● | - | - | - | No significant similarity found |
| ORF107e | 533,868 | 534,191 | 324 | ● | ● | - | - | - | No significant similarity found |
| ORF106a | 304,760 | 305,080 | 321 | ● | ● | 1 | 321 | 88.3 | hypothetical protein |
| ORF106b | 395,119 | 395,439 | 321 | ● | ○ | 1 | 321 | 87.9 | mitochondrial-like sequence |
| ORF105a | 23,648 | 23,965 | 318 | ● | ● | 264 | 317 | 94.4 | mitochondrial-like sequence |
| ORF105b | 337,204 | 337,521 | 318 | ○ | ○ | 164 | 287 | 76.2 | mitochondrial-like sequence |
| ORF105c | 410,439 | 410,756 | 318 | ○ | ○ | - | - | - | No significant similarity found |
| ORF104a | 144,361 | 144,675 | 315 | ● | ○ | - | - | - | No significant similarity found |
| ORF104b | 177,233 | 177,547 | 315 | ○ | ● | - | - | - | No significant similarity found |
| ORF103a | 191,936 | 192,247 | 312 | ● | ● | - | - | - | No significant similarity found |
| ORF103b | 218,110 | 218,421 | 312 | ○ | ● | 153 | 311 | 80.5 | mitochondrial-like sequence |
| ORF102a | 299,635 | 299,943 | 309 | ● | ● | 1 | 239 | 90.7 | hypothetical protein |
| ORF102b | 313,827 | 314,135 | 309 | ● | ● | 1 | 309 | 88.4 | mitochondrial-like sequence |
| ORF101 | 428,548 | 428,853 | 306 | ● | ● | 1 | 306 | 86.3 | hypothetical protein |
| ORF100a | 48,811 | 49,113 | 303 | ● | ○ | 1 | 303 | 72.4 | hypothetical protein |
| ORF100b | 85,135 | 85,437 | 303 | ● | ○ | - | - | - | No significant similarity found |
| ORF100c | 251,557 | 251,859 | 303 | ● | ● | 4 | 136 | 80.1 | mitochondrial-like sequence |
| ORF100d | 464,466 | 464,768 | 303 | ○ | ○ | 1 | 56 | 94.6 | mitochondrial-like sequence |
| ORF100e | 494,457 | 494,759 | 303 | ● | ● | 4 | 136 | 80.1 | mitochondrial-like sequence |
| ORF100f | 521,869 | 522,171 | 303 | ○ | ○ | 1 | 56 | 94.6 | mitochondrial-like sequence |
| ORF99 | 16,077 | 16,376 | 300 | ● | ○ | - | - | - | No significant similarity found |

We selected ORFs that overlaps at least 80% against transcriptome or nuclear genome. * indicates potential chimeric ORFs (see Table S6). ^a^Contigs in the draft nuclear genome were filtered with coverage cutoff of 90 % (i.e. percent of the contigs of nuclear genome sequences that overlaps the *Rhazya* mitochondrial sequences).

**Table S6.** Putative chimeric ORFs.

|  | ORF start | ORF end | ORF length | Identity | ORF hit start | ORF hit end | Chimera length | Gene hit start | Gene hit end | E-value | Gene | No of Transmembrane helices / probabilities |
| --- | --- | --- | --- | --- | --- | --- | --- | --- | --- | --- | --- | --- |
| ORF237a | 449,100 | 449,813 | 714 | 100 | 4 | 34 | 31 | 4 | 34 | 2.00E-10 | *sdh3* | 1 / 0.853 |
| ORF237b | 536,824 | 537,537 | 714 |  |  |  |  |  |  |  |  |  |
| ORF87 | 402,688 | 402,951 | 264 | 97.87 | 1 | 47 | 47 | 196 | 242 | 5.00E-18 | *atp9* | 0 / 0.146 |
| ORF75 | 315,539 | 315,766 | 228 | 96.67 | 19 | 48 | 30 | 392 | 363 | 7.00E-09 | *rpl2* | 0 / 0.505 |
| ORF73a | 257,432 | 257,653 | 222 | 100 | 67 | 195 | 129 | 265 | 393 | 3.00E-64 | *sdh4* | 2 / 0.753 |
| ORF73b | 500,332 | 500,553 | 222 |  |  |  |  |  |  |  |  |  |
| ORF72 | 140,522 | 140,740 | 219 | 93.55 | 84 | 114 | 31 | 31 | 1 | 2.00E-08 | *rrn26* | 0 / 0.512 |
| ORF69a | 257,425 | 257,634 | 210 | 100 | 74 | 202 | 129 | 265 | 393 | 3.00E-64 | *sdh4* | 0 / 0.120 |
| ORF69b | 500,325 | 500,534 | 210 |  |  |  |  |  |  |  |  |  |
| ORF59 | 507,849 | 508,028 | 180 | 96.77 | 140 | 170 | 31 | 710 | 680 | 2.00E-09 | *ccmFn* | 0 / 0.598 |
| ORF56a | 278,433 | 278,603 | 171 | 100 | 1 | 44 | 44 | 628 | 671 | 3.00E-18 | *rps4* | 0 / 0.621 |
| ORF56b | 507,783 | 507,953 | 171 | 91.07 | 1 | 55 | 55 | 1 | 56 | 1.00E-16 | *rpl2* | 1 / 0.942 |
|  |  |  |  | 92.31 | 45 | 83 | 39 | 1266 | 1304 | 4.00E-11 | *matR* |  |
|  |  |  |  | 96.77 | 77 | 107 | 31 | 680 | 710 | 2.00E-09 | *ccmFn* |  |

**Table S7.** Blast result of plastid-derived DNA segments in mitochondrial genome of Rhazya stricta.

|  | Query start | Query end | Mt-length | Aligned length | Pt-length | Hit start | Hit end | Identity | Bit-Score | E value | Anontation |
| --- | --- | --- | --- | --- | --- | --- | --- | --- | --- | --- | --- |
| 1 | 19,807 | 21,625 | 1,819 | 1,819 | 1,819 | 124,366 | 122,548 | 98.7 | 3,122.92 | 0.00E+00 | *rps15/ndhH IGS*,* ***ndhH****, ndhA exon1, ndhA intron** |
| 2 | 21,692 | 21,918 | 227 | 230 | 230 | 122,483 | 122,253 | 96.1 | 372.78 | 5.14E-102 | *ndhA intron** |
| 3 | 22,642 | 23,342 | 701 | 717 | 716 | 137,003 | 136,288 | 97.1 | 1,150.03 | 0.00E+00 | *trnI-GAU intron** |
| 4 | 30,530 | 31,309 | 780 | 809 | 801 | 29,372 | 30,172 | 92.0 | 1,108.55 | 0.00E+00 | *petN/psbM IGS** |
| 5 | 31,381 | 31,534 | 154 | 154 | 150 | 30,325 | 30,474 | 92.2 | 223.10 | 5.88E-57 | *petN/psbM IGS** |
| 6 | 31,519 | 31,920 | 402 | 412 | 385 | 30,820 | 31,204 | 88.3 | 508.03 | 9.91E-143 | *psbM/trnD-GUC IGS** |
| 7 | 33,734 | 33,976 | 243 | 243 | 242 | 36,070 | 36,311 | 97.1 | 403.44 | 3.04E-111 | *psbC** |
| 8 | 36,489 | 36,926 | 438 | 453 | 453 | 105,729 | 106,181 | 93.6 | 637.88 | 0.00E+00 | *trnA-UGC/rrn23 IGS*, rrn23** |
| 9 | 44,813 | 45,515 | 703 | 703 | 703 | 101,882 | 101,180 | 99.6 | 1,254.63 | 0.00E+00 | *rrn16/trnV-GAC IGS*,* ***trnV-GAC****, trnV-GAC/rps12 IGS** |
| 10 | 45,504 | 46,111 | 608 | 608 | 608 | 101,099 | 100,492 | 97.7 | 1,034.62 | 0.00E+00 | *trnV-GAC/rps12 IGS** |
| 11 | 58,772 | 58,919 | 148 | 155 | 155 | 113,076 | 112,922 | 92.3 | 228.51 | 1.38E-58 | *ndhF** |
| 12 | 121,944 | 122,010 | 76 | 73 | 73 | 88,173 | 88,245 | 83.6 | 78.83 | 1.58E-13 | *trnI-CAU** |
| 13 | 132,426 | 132,509 | 84 | 84 | 84 | 110,120 | 110,203 | 92.9 | 125.72 | 1.21E-27 | *trnR-ACC/trnN-GUU IGS*,* ***trnN-GUU****, trnN-GUU/ycf1 IGS** |
| 14 | 152,211 | 152,368 | 158 | 158 | 158 | 27,201 | 27,044 | 95.6 | 253.76 | 3.47E-66 | *rpoB** |
| 15 | 159,826 | 159,905 | 80 | 80 | 80 | 151,305 | 151,226 | 98.9 | 140.15 | 5.51E-32 | *ycf2** |
| 16 | 202,977 | 205,401 | 2,425 | 2,464 | 2,422 | 55,551 | 57,972 | 94.9 | 3,777.54 | 0.00E+00 | *atpB*, atpB/rbcL IGS, rbcL** |
| 17 | 209,331 | 209,405 | 75 | 75 | 75 | 71,986 | 71,912 | 97.3 | 127.52 | 3.48E-28 | *clpP intron2** |
| 18 | 221,891 | 221,970 | 80 | 80 | 80 | 25 | 104 | 97.5 | 136.54 | 6.71E-31 | *rps19/trnH-GUG*,* ***trnH-GUG****, trnH-GUG/psbA IGS* |
| 19 | 236,270 | 237,891 | 1,622 | 1,644 | 1,637 | 87,833 | 89,469 | 95.2 | 2,587.32 | 0.00E+00 | *rpl23*, rpl32/trnI-CAU IGS,* ***trnI-CAU****, trnI-CAU/ycf2 IGS, ycf2** |
| 20 | 282,006 | 283,743 | 1,738 | 1,791 | 1,779 | 16,772 | 14,994 | 93.5 | 2,569.28 | 0.00E+00 | *rps2*, rps2/atpl IGS,* ***atpI****, atpl/atpH IGS** |
| 21 | 283,727 | 283,922 | 196 | 202 | 184 | 14,881 | 14,698 | 86.1 | 224.90 | 1.68E-57 | *atpl/atpH IGS** |
| 22 | 283,992 | 285,680 | 1,689 | 1,725 | 1,693 | 14,636 | 12,944 | 91.8 | 2,381.73 | 0.00E+00 | *atpI/atpH IGS*,* ***atpH****, atpH/atpF IGS, atpF exon1, atpF intron** |
| 23 | 285,755 | 286,358 | 604 | 618 | 617 | 12,247 | 11,631 | 96.0 | 998.55 | 0.00E+00 | *atpF/atpA IGS*, atpA** |
| 24 | 397,695 | 397,803 | 109 | 109 | 109 | 31,584 | 31,476 | 94.5 | 170.80 | 3.25E-41 | *trnY/trnD IGS*,* ***trnD-GUC****, trnD-GUC/psbM IGS** |
| 25 | 398,379 | 398,517 | 262 | 262 | 262 | 46,411 | 46,549 | 96.4 | 228.51 | 2.16E-61 | *ycf3/trnS-GGA IGS*,* ***trnS-GGA*** |
| 26 | 398,726 | 399,285 | 560 | 566 | 550 | 46,768 | 47,317 | 82.3 | 524.26 | 6.08E-150 | *trnS-GGA/rps4 IGS*, rps4** |
| 27 | 438,492 | 443,560 | 5,069 | 5,069 | 5,062 | 38,588 | 43,649 | 99.7 | 9,041.57 | 0.00E+00 | *rps14/psaB IGS*,* ***psaB****, psaB/psaA IGS,* ***psaA****, psaA/ycf3 IGS** |
| 28 | 443,641 | 443,779 | 139 | 139 | 139 | 43,730 | 43,868 | 100.0 | 226.71 | 4.82E-58 | *psaA/ycf3 IGS** |
| 29 | 443,861 | 445,623 | 1,763 | 1,771 | 1,759 | 43,945 | 45,703 | 98.8 | 2,908.32 | 0.00E+00 | *psaA/ycf3 IGS*, ycf exon3, ycf3 intron2, ycf exon2, ycf3 intron1** |
| 30 | 449,060 | 449,171 | 112 | 119 | 119 | 154,180 | 154,298 | 87.4 | 145.56 | 1.30E-33 | *rpl2 intron*, rpl2 exon2** |
| 31 | 479,170 | 480,791 | 1,622 | 1,644 | 1,637 | 87,833 | 89,469 | 95.2 | 2,587.32 | 0.00E+00 | *rpl23*, rpl32/trnI-CAU IGS,* ***trnI-CAU****, trnI-CAU/ycf2 IGS, ycf2** |
| 32 | 512,500 | 513,254 | 755 | 789 | 756 | 65,572 | 66,327 | 80.2 | 708.21 | 0.00E+00 | *peA/psbJ IGS*,* ***psbJ****, psbJ/psbL IGS,* ***psbL****, psbL/psbF IGS,* ***psbF****, psbF/psbE IGS, psbE** |
| 33 | 513,299 | 513,434 | 136 | 137 | 137 | 68,031 | 68,167 | 89.8 | 181.62 | 1.43E-47 | *petG/trnW-CCA IGS*,* ***trnW-CCA****, trnW-CCA/trnP-UGG IGS** |
| 34 | 513,506 | 513,655 | 150 | 156 | 152 | 68,268 | 68,419 | 85.3 | 174.41 | 3.40E-45 | *trnW-CCA/trnP-UGG IGS*,* ***trnP-UGG****, trnP-UGG/psaJ IGS** |
| 35 | 537,466 | 537,577 | 112 | 119 | 119 | 154,298 | 154,180 | 87.4 | 145.56 | 1.30E-33 | *rpl2 exon2*, rpl2 intron** |
| 36 | 541,014 | 542,776 | 1,763 | 1,771 | 1,759 | 45,703 | 43,945 | 98.8 | 2,850.61 | 0.00E+00 | *ycf3 intron1*, ycf exon2, ycf3 intron2, ycf exon3, psaA/ycf3 IGS** |
| 37 | 542,858 | 542,996 | 139 | 139 | 139 | 43,868 | 43,730 | 100.0 | 226.71 | 4.82E-58 | *ycf3/psaA IGS** |
| 38 | 543,077 | 548,145 | 5,069 | 5,069 | 5,062 | 43,649 | 38,588 | 99.7 | 9,041.57 | 0.00E+00 | *ycf3/psaAIGS*,* ***psaA****, psaB/psaA IGS,* ***psaB****, psaB/rps14 IGS*,* |

Bold fonts indicate intact genes, * indicates fragments, red colors indicate pseudogene, green colors indicate second copy in repeats.

**Table S8.** Predicted RNA editing in 38 protein-coding genes for Rhazya mitochondrial genome.

| protein  genes |  | PREP-Mt | | | PREPACT v2.0 | | |
| --- | --- | --- | --- | --- | --- | --- | --- |
|  | cutoff value | 1 | 0.6 | **0.5** | 100% | 75% | 50% |
|  | CDS length |  |  |  |  |  |  |
| *atp1* | 1530 | 2 | 5 | **6** | 1 | 5 | 7 |
| *atp4* | 597 | 4 | 6 | **9** | 1 | 5 | 7 |
| *atp6* | 774 | 14 | 19 | **19** | 4 | 17 | 19 |
| *atp8* | 474 | 1 | 2 | **2** | 1 | 2 | 3 |
| *atp9* | 273 | 8 | 8 | **8** | 3 | 8 | 8 |
| *ccmB* | 621 | 15 | 31 | **31** | 2 | 26 | 32 |
| *ccmC* | 753 | 10 | 27 | **27** | 1 | 13 | 26 |
| *ccmFc* | 1308 | 11 | 16 | **17** | 0 | 15 | 22 |
| *ccmFn* | 1711 | 19 | 29 | **31** | 1 | 22 | 29 |
| *cob* | 1182 | 8 | 10 | **10** | 6 | 9 | 10 |
| *cox1* | 1584 | 15 | 17 | **17** | 7 | 16 | 17 |
| *cox2* | 768 | 7 | 8 | **8** | 4 | 7 | 8 |
| *cox3* | 798 | 2 | 9 | **9** | 1 | 8 | 9 |
| *matR* | 1980 | 7 | 12 | **12** | 2 | 12 | 18 |
| *mttB* | 837 | 7 | 23 | **27** | 0 | 18 | 28 |
| *nad1* | 1026 | 10 | 16 | **16** | 0 | 13 | 15 |
| *nad2* | 1467 | 16 | 23 | **24** | 3 | 17 | 24 |
| *nad3* | 357 | 8 | 14 | **14** | 3 | 13 | 13 |
| *nad4* | 1488 | 26 | 38 | **38** | 2 | 34 | 39 |
| *nad4L* | 303 | 8 | 14 | **14** | 1 | 12 | 14 |
| *nad5* | 2013 | 9 | 23 | **23** | 5 | 19 | 24 |
| *nad6* | 618 | 6 | 9 | **9** | 0 | 5 | 8 |
| *nad7* | 1185 | 18 | 24 | **24** | 9 | 24 | 24 |
| *nad9* | 573 | 3 | 6 | **6** | 3 | 6 | 6 |
| *rpl2* | 987 | 2 | 3 | **3** | 0 | 2 | 5 |
| *rpl5* | 555 | 4 | 7 | **7** | 0 | 6 | 7 |
| *rpl10* | 489 | 0 | 3 | **3** | 0 | 0 | 1 |
| *rpl16* | 516 | 3 | 5 | **5** | 0 | 5 | 6 |
| *rps1* | 597 | 1 | 1 | **1** | 0 | 2 | 2 |
| *rps3* | 1692 | 2 | 7 | **8** | 1 | 4 | 8 |
| *rps4* | 1047 | 6 | 13 | **14** | 0 | 7 | 15 |
| *rps7* | 447 | 0 | 2 | **2** | 0 | 1 | 2 |
| *rps10* | 423 | 3 | 4 | **4** | 3 | 3 | 4 |
| *rps12* | 378 | 1 | 3 | **3** | 0 | 3 | 3 |
| *rps13* | 351 | 1 | 4 | **4** | 3 | 3 | 5 |
| *rps14* | 279 | 0 | 1 | **1** | 0 | 1 | 1 |
| *rps19* | 285 | 2 | 2 | **2** | 1 | 1 | 4 |
| *sdh4* | 459 | 0 | 4 | **4** | 0 | 2 | 7 |

**Table S9.** RNA editing validation of 11 genes using transcriptome data.

| Genes | position | Tc | > 0.5 | Genes | position | Tc | > 0.5 | Genes | position | Tc | > 0.5 |
| --- | --- | --- | --- | --- | --- | --- | --- | --- | --- | --- | --- |
| *atp1* | 7 |  | - | *nad4* | **154** |  | 0.97 | *nad5* | **1895** |  | 0.69 |
|  | **1039** |  | 0.98 |  | **158** |  | 0.90 |  | **1916** |  | 0.75 |
|  | **1178** |  | 1.00 |  | **166** |  | 0.90 |  | **1918** |  | 0.84 |
|  | **1292** |  | 0.99 |  | **197** |  | 0.96 |  | **1958** |  | - |
|  | **1415** |  | 0.95 |  | **362** |  | 0.98 | *nad7* | **38** |  | 1.00 |
|  | **1490** |  | 1.00 |  | **368** |  | 0.85 |  | **77** |  | 1.00 |
| *atp9* | **20** |  | 1.00 |  | **376** |  | 0.90 |  | **83** |  | 1.00 |
|  | - | 81 | 0.80 |  | **416** |  | 0.92 |  | **137** |  | 1.00 |
|  | **82** |  | 1.00 |  | **433** |  | - |  | **200** |  | 1.00 |
|  | **92** |  | 1.00 |  | **436** |  | 0.73 |  | **209** |  | 0.95 |
|  | **182** |  | 1.00 |  | **437** |  | - |  | **251** |  | 0.99 |
|  | **191** |  | 0.73 |  | **577** |  | - |  | **316** |  | 0.85 |
|  | - | 205 | 0.84 |  | **608** |  | 1.00 |  | **335** |  | 0.93 |
|  | **212** |  | 0.85 |  | **659** |  | 0.98 |  | **344** |  | 0.85 |
|  | **215** |  | 1.00 |  | **767** |  | 0.99 |  | **383** |  | 0.98 |
|  | **223** |  | 1.00 |  | **836** |  | 0.98 |  | **533** |  | 0.67 |
| *cox1* | **11** |  | 1.00 |  | **857** |  | 0.99 |  | **578** |  | 0.99 |
|  | - | 15 | 0.53 |  | **887** |  | 1.00 |  | **724** |  | 0.95 |
|  | **242** |  | 0.99 |  | **956** |  | - |  | **734** |  | 0.99 |
|  | **254** |  | 1.00 |  | **977** |  | - |  | **739** |  | 0.54 |
|  | **443** |  | 0.99 |  | **1010** |  | 0.98 |  | **740** |  | 0.71 |
|  | **452** |  | 1.00 |  | **1016** |  | 1.00 |  | **769** |  | 0.93 |
|  | **515** |  | 1.00 |  | - | 1101 | 0.71 |  | **926** |  | 0.89 |
|  | **551** |  | 1.00 |  | **1109** |  | 0.97 |  | **944** |  | 0.89 |
|  | **590** |  | 1.00 |  | **1129** |  | 0.81 |  | - | 963 | 0.80 |
|  | **668** |  | 1.00 |  | **1132** |  | 0.59 |  | - | 1050 | 0.98 |
|  | **715** |  | 1.00 |  | **1148** |  | 0.91 |  | **1057** |  | 1.00 |
|  | **761** |  | 0.96 |  | **1151** |  | 0.89 |  | **1103** |  | 1.00 |
|  | **1037** |  | 1.00 |  | **1172** |  | 0.96 |  | **1124** |  | 1.00 |
|  | **1186** |  | 0.99 |  | **1355** |  | 0.94 |  | **1166** |  | 0.94 |
|  | **1405** |  | 0.99 |  | **1373** |  | 1.00 | *rpl5* | **35** |  | 0.94 |
|  | **1433** |  | 1.00 |  | **1405** |  | 0.94 |  | **47** |  | 0.93 |
|  | **1489** |  | 1.00 |  | **1417** |  | 0.85 |  | **64** |  | 0.96 |
|  | **1499** |  | 1.00 |  | **1433** |  | 1.00 |  | **92** |  | 0.97 |
| *cox2* | **379** |  | 0.50 |  | **1438** |  | 0.95 |  | - | 161 | 0.92 |
|  | **443** |  | 0.75 | *nad5* | **155** |  | 0.86 |  | **163** |  | 0.94 |
|  | **461** |  | 1.00 |  | **242** |  | 1.00 |  | **509** |  | 0.99 |
|  | **476** |  | 0.94 |  | **358** |  | 1.00 |  | **512** |  | 0.98 |
|  | **544** |  | 1.00 |  | **359** |  | 0.75 | *rps4* | 38 |  | - |
|  | **557** |  | 1.00 |  | **374** |  | 0.98 |  | **164** |  | 0.94 |
|  | **632** |  | 1.00 |  | **398** |  | 0.99 |  | **193** |  | 0.83 |
|  | **742** |  | 0.86 |  | **539** |  | 1.00 |  | **266** |  | 0.81 |
| *cox3* | **298** |  | 0.93 |  | - | 548 | 1.00 |  | **278** |  | 0.94 |
|  | **311** |  | - |  | - | 608 | 0.93 |  | **290** |  | 0.96 |
|  | **314** |  | 0.95 |  | **629** |  | 1.00 |  | **335** |  | 0.96 |
|  | **419** |  | 0.96 |  | **676** |  | 0.97 |  | **482** |  | 0.90 |
|  | **422** |  | 0.92 |  | **713** |  | 0.94 |  | **914** |  | 1.00 |
|  | **512** |  | 0.92 |  | **725** |  | 0.98 |  | **925** |  | 1.00 |
|  | **566** |  | 0.94 |  | **835** |  | 0.99 |  | **935** |  | 0.94 |
|  | **754** |  | 0.97 |  | **1310** |  | 0.98 |  | **950** |  | 1.00 |
|  | **764** |  | 0.87 |  | **1490** |  | 0.99 |  | **1001** |  | 0.97 |
| *nad4* | **29** |  | 0.94 |  | **1550** |  | 0.81 |  | **1015** |  | 0.88 |
|  | **74** |  | - |  | **1568** |  | 0.58 | *rps7* | **116** |  | 0.74 |
|  | **77** |  | 1.00 |  | **1580** |  | - |  | **332** |  | 0.86 |
|  | - | 84 | 0.95 |  | **1589** |  | 0.63 |  |  |  |  |
|  | **107** |  | 1.00 |  | **1610** |  | 0.91 |  |  |  |  |

The number of positions indicate predicted RNA editing sites by PREP-Mt with cutoff value of 0.5 (bold indicate cutoff value of 0.6). Tc indicate predicted RNA editing sites by transcriptome data (> 0.5 indicate the cutoff value of 50% coverage).

**Table S10.** Genome size, GC content, repetitive DNA, plastid-like DNA, and transposable elements in eight asterid mitochondrial genomes.

|  | ***Asclepias*** | ***Rhazya*** | ***Nicotiana*** | ***Boea*** | ***Mimulus*** | ***Daucus*** | ***Helianthus*** | ***Vaccinium*** |
| --- | --- | --- | --- | --- | --- | --- | --- | --- |
| **Genome size (bp)** | 682,498 | 548,608 | 430,597 | 510,519 | 525,671 | 281,132 | 300,945 | 459,678 |
| **GC content (%)** | 43.40 | 43.70 | 45.00 | 43.30 | 45.10 | 45.40 | 45.00 | 45.30 |
| **Repetitive DNA (bp)** | 8,116 | 77,887 | 34,532 | 5,038 | 41,125 | 54,514 | 19,357 | 22,192 |
| Total (%) | 1.2 | 14.2 | 8.0 | 1.0 | 7.8 | 19.4 | 6.4 | 4.8 |
| <50 | 12 (0.08) | 19 (0.16) | 16 (0.16) | 5 (0.04) | 13 (0.12) | 18 (0.28) | 11 (0.16) | 308 (2.06) |
| 50-99 | 25 (0.24) | 49 (0.59) | 50 (0.79) | 27 (0.35) | 38 (0.46) | 79 (1.90) | 28 (0.67) | 58 (0.88) |
| 100-499 | 18 (0.58) | 15 (0.45) | 36 (1.23) | 14 (0.42) | 15 (0.51) | 22 (1.03) | 15 (1.06) | 21 (0.77) |
| 500-1999 | 2 (0.28) | 1 (0.37) | 0 (0.00) | 2 (0.45) | 0 (0.00) | 0 (0.00) | 1 (0.24) | 6 (1.12) |
| ≥2000 | 0 (0.00) | 2 (12.62) | 3 (9.11) | 0 (0.00) | 4 (7.82) | 4 (24.61) | 1 (4.29) | 0 (0.00) |
| **Plastid-derived DNA (bp)** | 47,259 | 32,871 | 10,703 | 31,433 | 17,149 | 7,576 | 4,966 | 7,683 |
| (%) | 6.9 | 6.0 | 2.5 | 6.2 | 3.3 | 2.7 | 1.7 | 1.7 |
| **Transposable elements (bp)** | 20,919 | 16,008 | 18,817 | 21,787 | 18,632 | 11,674 | 9,253 | 27,403 |
| (%) | 3.1 | 2.9 | 4.4 | 4.3 | 3.5 | 4.2 | 3.1 | 6.0 |
| DNA-transposon | 3,274 | 2,331 | 2,749 | 3,115 | 1,740 | 1,405 | 1,865 | 1,742 |
| LTR-retrotransopon | 16,296 | 12,604 | 14,869 | 16,184 | 14,873 | 9,523 | 6,839 | 20,910 |
| *copia*-like | 10,153 | 5,859 | 8,096 | 8,436 | 5,739 | 1,675 | 1,648 | 11,599 |
| *gypsy*-like | 6,027 | 6,745 | 6,773 | 7,567 | 9,134 | 7,848 | 5,191 | 8,293 |
| Non-LTR retrotransposon | 1,349 | 1,073 | 1,199 | 2,488 | 2,019 | 746 | 549 | 4,751 |

**Table S11.** Rhazya rRNA and tRNA content compared to other asterids and two other representative angiosperms.

|  | *Rhazya* | *Asclepias* | *Nicotiana* | *Mimulus* | *Boea* | *Daucus* | *Helianthus* | *Vaccinium* | *Silene* | *Arabidopsis* |
| --- | --- | --- | --- | --- | --- | --- | --- | --- | --- | --- |
| **Ribosomal RNA** | | | | | | | | | | |
| *rrn26* | ● | ● | ● | ● | ● | ● | ● | ● | ● | ● |
| *rrn18* | ● | ● | ● | ● | ● | ● | ● | ● | ● | ● |
| *rrn5* | ● | ● | ● | ● | ● | ● | ● | ● | ● | ● |
| **tRNA genes derived from mitochondrial origin** | | | | | | | | | | |
| *trnC-GCA* | ● | ● | ● | ● | ● | ● | ● | ● | ● | ● |
| *trnD-GUC* | ○ | ○ | ψ | ○ | ○ | ○ | ○ | ○ | ○ | ○ |
| *trnE-UUC* | ● | ● | ● | ● | ● | ● | ● | ● | ● | ● |
| *trnF-GAA* | ● | ● | ● | ● | ● | ● | ● | ● | ○ | ● |
| *trnG-GCC* | ● | ● | ● | ● | ● | ● | ● | ○ | ○ | ● |
| *trnI-CAU* | ● | ● | ● | ● | ● | ● | ● | ● | ● | ● |
| *trnK-UUU* | ● | ● | ● | ● | ● | ● | ● | ● | ○ | ● |
| *trnfM-CAU* | ● | ● | ● | ● | ● | ● | ● | ● | ● | ● |
| *trnP-UGG* | ● | ● | ● | ● | ● | ● | ● | ● | ● | ● |
| *trnQ-UUG* | ● | ● | ● | ● | ● | ● | ● | ● | ○ | ● |
| *trnS-GCU* | ● | ● | ● | ● | ● | ● | ● | ● | ○ | ● |
| *trnS-UGA* | ● | ● | ● | ● | ● | ● | ○ | ● | ○ | ● |
| *trnY-GUA* | ● | ● | ● | ● | ● | ● | ● | ● | ● | ● |
| **tRNA genes derived from plastid origin** | | | | | | | | | | |
| ***trnD-GUC**** | ● | ● | ● | ● | ● | ● | ● | ● | ○ | ● |
| *trnE-UUC* | - | - | ● | - | - | - | - | - | - | - |
| *trnF-GAA* | - | ● | - | ● | ● | - | - | - | - | - |
| ***trnH-GUG**** | ● | ● | ● | ● | ● | ● | ● | - | ● | ● |
| *trnI-CAU* | ●^x2^, ψ | ● | ● | - | - | ● | ● | ψ | - | - |
| *trnL-CAA* | - | ● | - | - | ● | - | - | ● | - | - |
| ***trnM-CAU**** | ○ | ● | ● | ● | ● | ○ | ● | ● | ψ | ● |
| ***trnN-GUU**** | ● | ● | ● | ● | ● | ● | ● | ● | ● | ● |
| *trnP-UGG* | ψ | - | ● | - | - | - | - | - | ψ | - |
| *trnR-ACG* | - | - | - | - | ● | - | - | - | - | - |
| ***trnS-GGA**** | ● | ● | ● | ● | ● | ○ | ○ | - | ○ | ● |
| *trnV-GAC* | ● | - | - | - | ● | - | ● | - | - | - |
| *trnW-CCA* | ● | ● | ● | ● | ● | ● | ● | ● | ● | ● |

●; present, ○; loss, -; absent, ψ; pseudogene. *; the transferred plastid tRNA genes into the mitochondrial genome in the common ancestor of extant angiosperms [45,46].

**Table S12.** Primers used for testing alternative recombinogenic conformations and for confirming ORFs that represented (R)-mandelonitrile lyase gene in the Rhazya mitochondrial genome.

|  |  | Primer name | Sequence (5' → 3') |
| --- | --- | --- | --- |
| *Alternative*  *recombinogenic conformations* | | *Rhazya1* | GGA AGC CCA AGC CCT AGT TT |
|  |  | *Rhazya2* | CTC TTC TTT CGG CCG GGA AA |
|  |  | *Rhazya3* | AAT TGC GAG AAG GAA GCG GA |
|  |  | *Rhazya4* | TTC GTT TAC CGG GTT CAG CA |
|  |  | *Rhazya5* | GCT GCT TCT TTG ACC TGT GC |
|  |  | *Rhazya6* | TAG GCC CCA TTG CTG GAA AG |
|  |  | *Rhazya7* | CCC AGC TAC CGC TAT CCT TG |
|  |  | *Rhazya8* | TGT ATC ACG AGC GCA CTA CC |
|  |  | *Rhazya9* | TCG CGC CAT CTT CCT AAG AC |
|  |  | *Rhazya10* | CAC TAA CTC TGC CTG GGG TG |
|  |  | *Rhazya11* | CTT CGG GCT GAG GTC CAA TT |
|  |  | *Rhazya12* | CAC AC CGCT GGC AAA TTC AA |
|  |  | *Rhazya13* | TCC GTT CTT CCC AGC CAA TC |
|  |  | *Rhazya14* | GCA GGC AAA GGC TCC TTA GA |
| *RT-PCR* | *positive* | *RMDL_RT* | CGG AGA GGG AGA ACT CCT AA |
|  |  | *RMDL156F* | TGG TTT CTT TGC CCA CCG TA |
|  |  | *RMDL393F* | CAT AGT CGG AGG TGG CAC TG |
|  |  | *RMDL476R* | AAA GAG GCG GCG TTC CAT AT |
|  |  | *RMDL1343R* | CAC CAT GAG ACG GGG CAT AA |
|  | *negative* | *ccmFn_RT* | GGT TCC TGA GAC ACA GCA TG |
|  |  | *ccmFn295F* | GGT CGA CCC CAA AGC CAT AA |
|  |  | *ccmFn1391R* | GCT GTG GCT AAT ACC CGA GG |

**Table S13**. Information of phylogenetic analyses and alignment of *rps14* and *sdh3* genes.

| Group |  | Order | Species | *rps14* | *sdh3* |
| --- | --- | --- | --- | --- | --- |
|  |  | Amborellales | *Amborella trichopoda* (mt) | KF754803 | KF754803 |
|  | magnoliids | Magnoliales | *Liriodendron tulipifera* (mt) | NC_021152 | NC_021152 |
| monocots | commelinids | Arecales | *Phoenix dactylifera* (mt) | NC_016740 | - |
|  |  | Poales | *Juncus effusus* (mt) | DQ_380467 | - |
| eudicots | rosids | Vitales | *Vitis vinifera* (mt) | NC_012119 | NC_012119 |
|  |  | Cucurbitales | *Citrullus lanatus* (mt) | - | NC_014043 |
|  |  |  | *Cucumis sativus* (mt) | - | NC_016004 |
|  |  |  | *Cucurbita pepo* (mt) | - | NC_014050 |
|  |  | Malpighiales | *Ricinus communis* (mt) | - | NC_015141 |
|  |  | Fabales | *Glycine max* (mt) | NC_020455 | - |
|  |  |  | *Vigna radiata* (mt) | NC_015121 | - |
|  |  | Malvales | *Gossypium hirsutum* (mt) | - | AF362739 |
|  |  | Brassicales | *Arabidopsis thaliana* (n) | NC_003071 | NM_120997 |
|  |  |  | *Arabidopsis thaliana* (mt) | NC_001284 | - |
|  |  |  | *Brassica napus (mt)* | NC_008285 | - |
|  |  |  | *Carica papaya* (mt) | - | NC_012116 |
|  |  | Caryophyllales | *Silene latifolia* (n) | - | *FZQN^2008574^ |
|  |  |  | *Silene latifolia* (mt) | NC_014487 | NC_014487 |
|  |  |  | *Spergularia media* (n) | *TJES^2066465^ | - |
|  | asterids | Ericales | *Ledum palustre* (n) | *WXVX^2014809^ | *WXVX^2008168^ |
|  |  | Gentianales | *Asclepias syriaca* (n) | *YADI^2063334^ | - |
|  |  |  | *Asclepias syriaca* (mt) | - | NC_022796 |
|  |  |  | *Rhazya stricta1* (n) | KJ485851 | - |
|  |  |  | *Rhazya stricta2* (n) | KJ485852 | - |
|  |  |  | *Rhazya stricta* (n) | - | KJ485853 |
|  |  |  | *Rhazya stricta* (mt) | KJ485850 | KJ485850 |
|  |  |  | *Strychnos spinosa* (n) | - | *GGJD^2001979^ |
|  |  | Solanales | *Nicotiana tabacum* (mt) | NC_006581 | NC_006581 |
|  |  |  | *Solanum ptychanthum* (n) | *DLJZ^2052313^ | - |
|  |  | Lamiales | *Ajuga reptans* (n) | *UCNM^2009956^ | - |
|  |  |  | *Oxera pulchella* (n) | *RTNA^2015260^ | *GNPX^2004484^ |
|  |  |  | *Boea hygrometrica* (mt) | NC_016741 | NC_016741 |
|  |  |  | *Mimulus guttatus* (mt) | NC_018041 | - |
|  |  | Asterales | *Platycodon grandiflorus* (n) | *IHPC^2070391^ | - |
|  |  |  | *Lobelia siphilitica* (n) | - | *IZLO^2001202^ |
|  |  | Apiales | *Angelica archangelica* (n) | *TQKZ^2002624^ | - |
|  |  |  | *Heracleum lanatum* (n) | - | *CWYJ^2004062^ |

*The 1000 plants (1KP) database [https://sites.google.com/a/ualberta.ca/onekp/] was searched using Blast with nuclear-encoded *rps14* and *sdh3* gene. mt = mitochondrial, n = nuclear. The others indicate the NCBI accession numbers of sequences of individual *rps14* and *sdh3* genes or entire mitochondrial genomes from which gene sequences were extracted.
