# Supplementary material for: Complete sequences of organelle genomes from the medicinal plant Rhazya stricta (Apocynaceae) and contrasting patterns of mitochondrial genome evolution across asterids
Source: BMC Genomics. 2014 May 28;15(1):405. doi: 10.1186/1471-2164-15-405 (PMC4045975; doi:10.1186/1471-2164-15-405)
Supplement: Supplementary file 2 — Additional file 2: Figure S1: Seven families of repeats (R1-R7) of Rhazya mitochondrial genome involved in recombination. Figure S2. PCR strategy for identifying intramolecular recombination across mitochondrial repeats. Figure S3. The two mitochondrial ORFs representing the nuclear (R)-mandelonitrile lyase sequence [82]. Figure S4. Potential chimeric ORFs. Figure S5. Conserved mitochondrial gene blocks among eight asterids. (PDF 4 MB) [file 12864_2014_6093_MOESM2_ESM.pdf]

Figure S1. Seven families of repeats (R1-R7) of *Rhazya* mitochondrial genome involved in recombination. A. Illustration of corrected PacBio reads mapped against the assembled *Rhazya* mitochondrial genome. Blue and red lines indicate large and small repeat regions, respectively. Grey lines indicate regions of PacBio reads not aligned to the mitochondrial genome. **B.** Seven repeat families of *Rhazya* plotted on the mitochondrial genome. Blue lines within the circle indicate the positions of the pairs of repeats, twisted connecting lines denote repeats in reverse orientation. The letters A through F on the map indicated seven single-copy regions separated by repeats.

Figure S2. PCR strategy for identifying intramolecular recombination across mitochondrial repeats. Red lines on the outside of the master circle indicate the position and orientation of PCR primers to identify mitochondrial recombination, relative to master circle. Blue (direct repeats) or yellow (inverted repeats) lines on the inside of the master circle indicate the position and orientation of PCR primers to detect mitochondrial recombination; matched color (blue or yellow) lines on the outside of the eight alternative circles indicate alternative orientations of primers/products. Each repeat (R2; orange, R6; purple, and R7; green) or repeat cluster (R2-R3-R4; black) is indicated by an arrowhead on the circles and double-headed arrows show six possible alternative arrangements. Individual primer sequences are indicated by the numbers one through 12 and correspond to Table S12. The letters A through F on the maps indicate seven single-copy regions separated by repeats.

Figure S3. The two mitochondrial ORFs representing the nuclear (R)-mandelonitrile lyase sequence. **A.** Schematic diagram of the (R)-mandelonitrile lyase sequence in the *Rhazya* nucleus (nDNA; red line), the mitochondrial fragments of 247 and 1,894 bp, with the two ORFs (blue block arrows) in the *Rhazya* mitochondria (mtDNA; blue line), and the transcript (orange). A TE (transposable element) insertion is shown in red between the two fragments in the *Rhazya* mitochondrial DNA. The model (malate:quinone oxidoreductase; *Mqo* and

glucose-methanol-choline (GMC) oxidoreductase; *GMC_oxred_C*) of a putative nuclear protein-coding gene was predicted using the Conserved Domain Architecture Retrieval Tool (CDART) [82]. **B.** The broken outlines represent the full-length nuclear sequence, the red shaded arrow within indicates the proportion of the sequence present in six asterid mitochondrial genomes with the length of each indicated above. **C.** Nucleotide sequence alignment of the nuclear and mitochondrial (ORF185 and ORF430) copies and the mitochondrial transcript from *Rhazya stricta*. Red box shows 5 bp deletion that caused a frame shift to generate split ORFs. **D.** Amino acid sequence alignment of nuclear and two mitochondrial ORFs. Each ORF (blue; ORF185, red; ORF430) contains a conserved domain (*Mqo*; blue shaded box and *GMC_oxred_C*; red shaded box).

Figure S4. Potential chimeric ORFs. Schematic diagram of the genomic regions surrounding the ORFs containing repeats. **A.** ORF56b overlaps with repeat region 6 (R6) associated with *rpl2* and contains small fragments of three mitochondrial genes (*rpl2*, *matR,* and *ccmFn*). **B.** ORF318 and ORF324 were identified in repeat cluster regions associated with *atp6*. Repeats are numbered and indicated by solid dark blue arrows and are diagrammed on the mitochondrial genome map. The maps are broken (-//-) to focus on regions of interest. ORFs are indicated by light blue block arrows. Red bands on the ORFs indicate the transmembrane helices regions.

Figure S5. Conserved mitochondrial gene blocks among eight asterids. All pairwise comparisons were made relative to *R. stricta*. All protein-coding genes and rRNAs are arranged from top to bottom for *Rhazya*, and from left to right for the other asterids based on the order in published genome maps. Red genes names indicate losses of each mitochondrial genome. Blue squares represent the presence of a given gene in each genome. Red boxes indicate colinear gene blocks between two genomes. Shaded red boxes indicate shared colinear gene blocks among the eight asterid genomes.

Figure S1

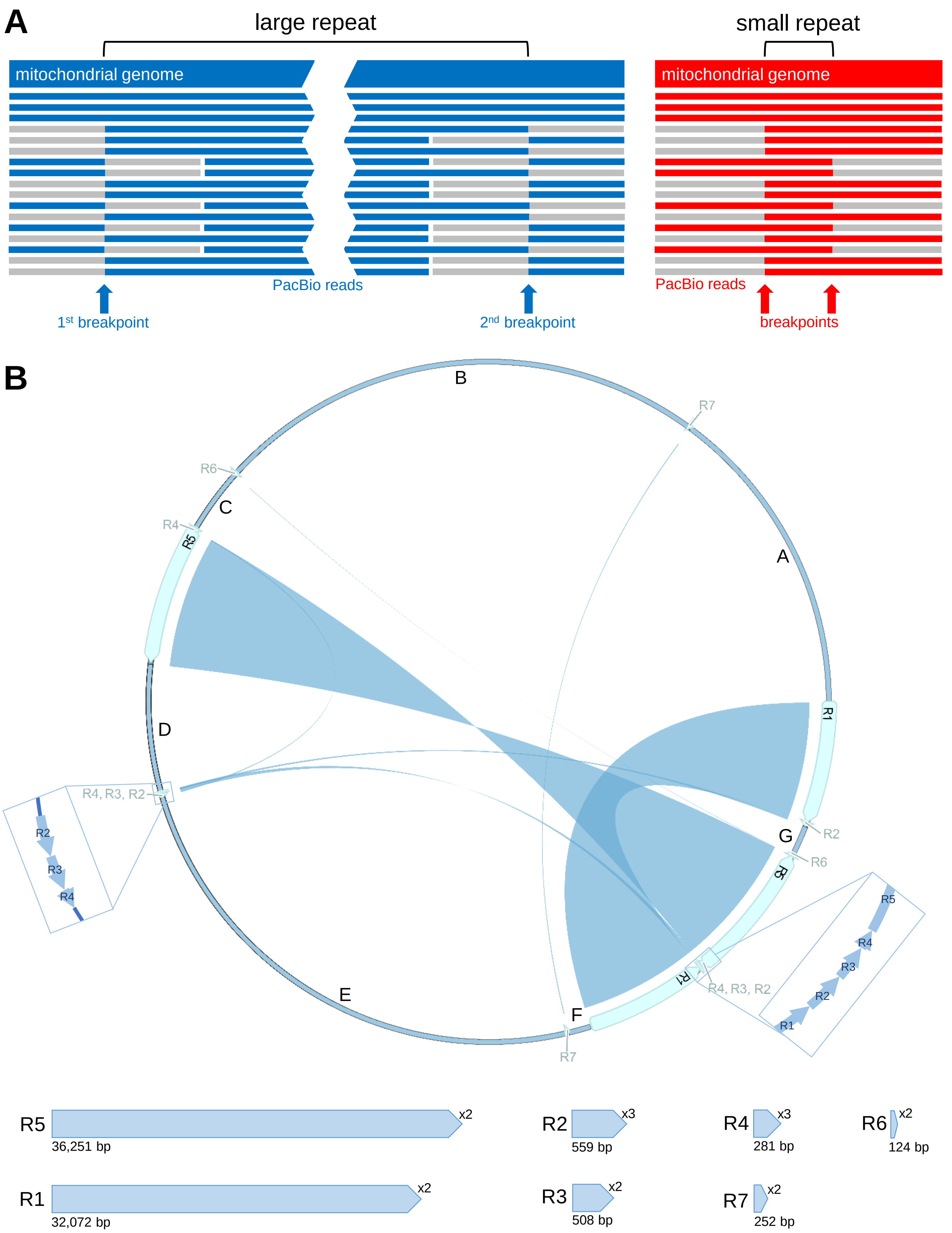
A
large repeat
small repeat
mitochondrial genome
mitochondrial genome
PacBio reads
PacBio reads
1st breakpoint
2nd breakpoint
breakpoints
B
A
B
C
D
E
F
G
R1
R2
R3
R4
R5
R6
R7
R4, R3, R2
R5 36,251 bp x2
R1 32,072 bp x2
R2 559 bp x3
R3 508 bp x2
R4 281 bp x3
R7 252 bp x2
R6 124 bp x2

Figure S2

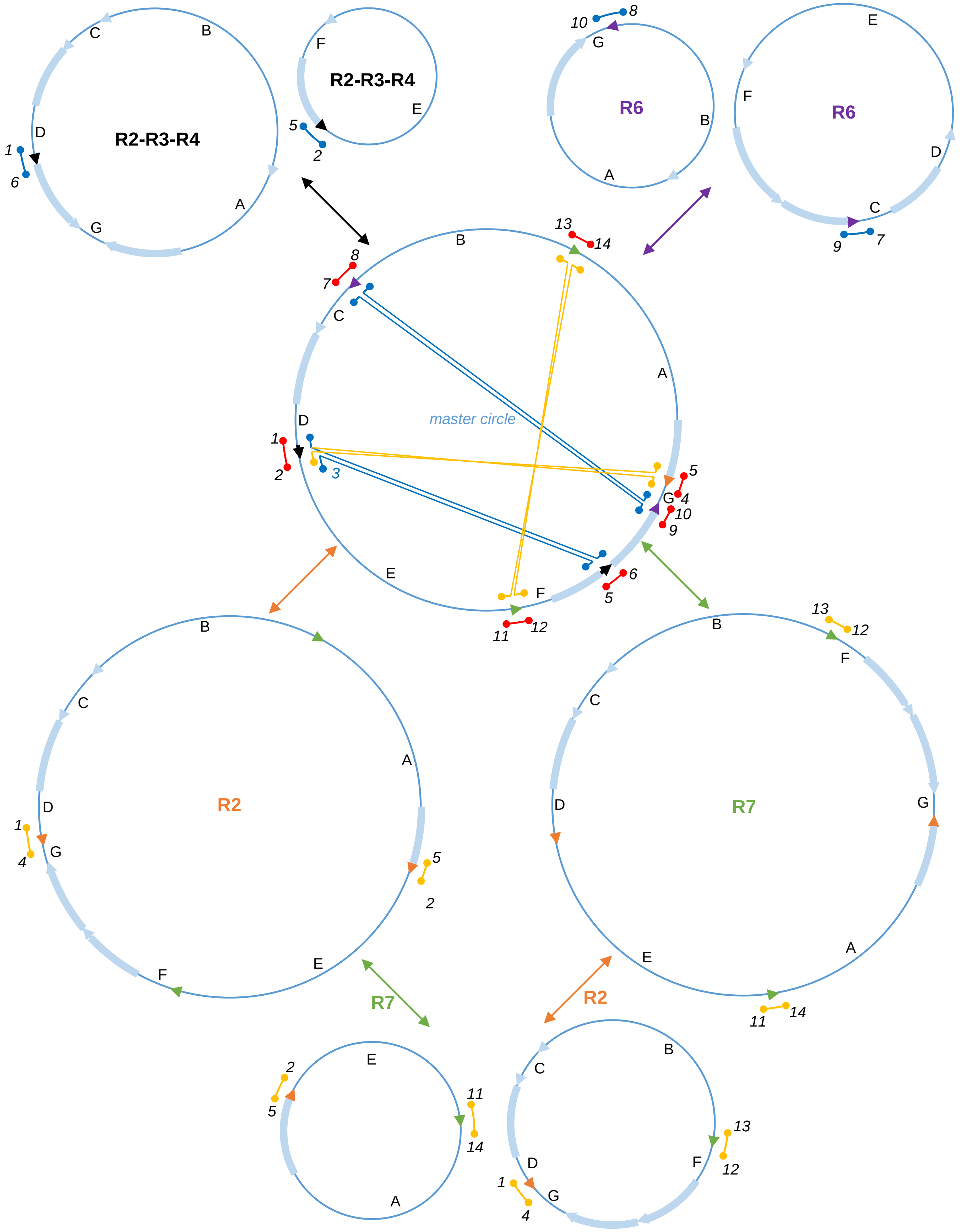

R2-R3-R4
C
B
D
A
G
1
6
F
E
5
2
R6
G
B
A
10
8
E
F
D
C
9
7
master circle
13
14
8
7
C
A
D
1
2
3
5
4
10
9
6
5
E
F
11
12
R2
B
C
A
D
G
5
2
F
E
R7
13
12
B
F
C
G
D
A
E
11
14
R7
E
2
5
11
14
A
R2
B
C
13
12
F
D
1
G
4

Figure S3

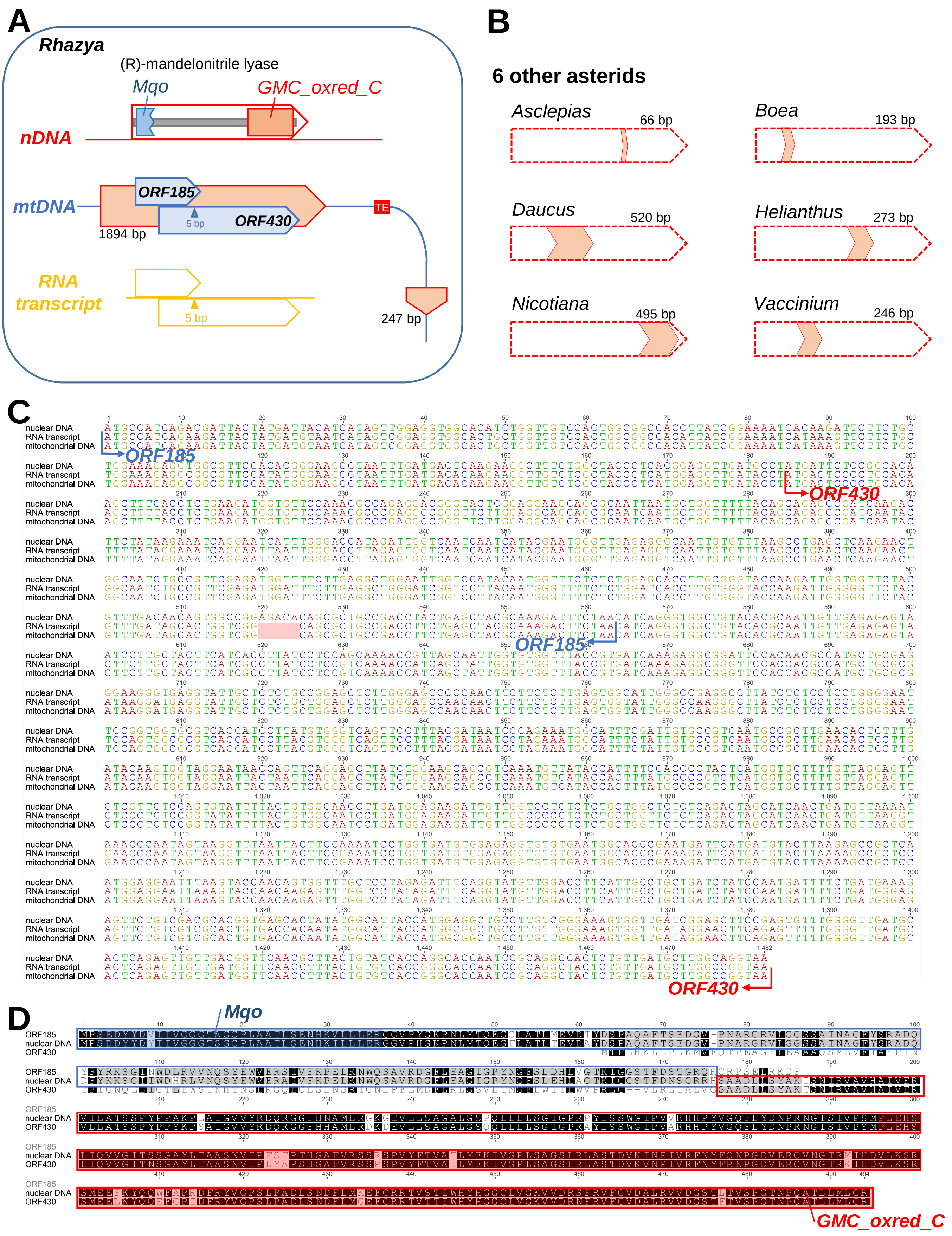

A
Rhazya
(R)-mandelonitrile lyase
Mqo
GMC_oxred_C
nDNA
mtDNA
ORF185
ORF430
5 bp
1894 bp
TE
247 bp
RNA transcript
B
6 other asterids
Asclepias
66 bp
Boea
193 bp
Daucus
520 bp
Helianthus
273 bp
Nicotiana
495 bp
Vaccinium
246 bp
C
nuclear DNA
RNA transcript
mitochondrial DNA
ORF185
ORF430
D
Mqo
ORF185
nuclear DNA
ORF430
GMC_oxred_C

Figure S4

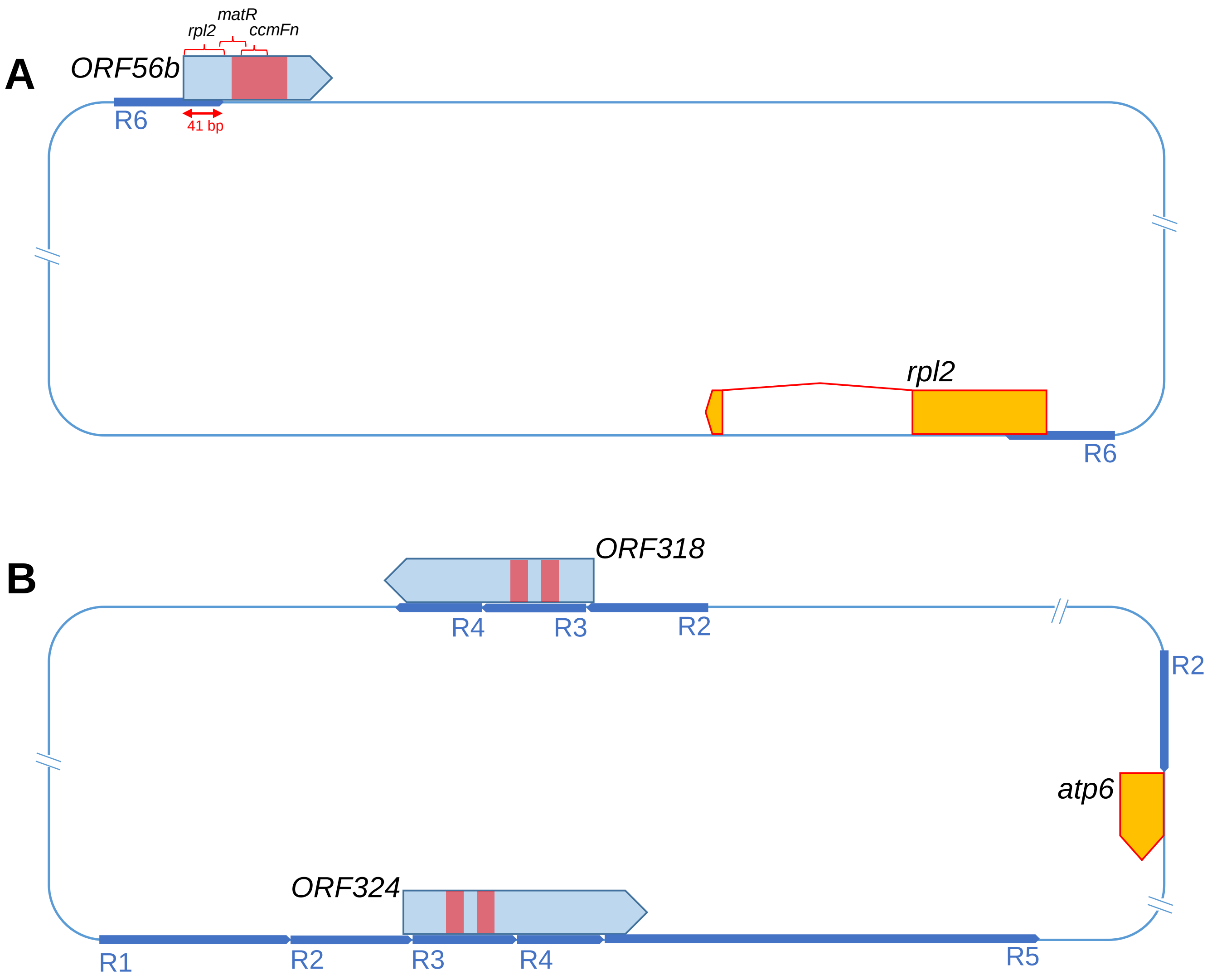
A
ORF56b
rpl2
matR
ccmFn
R6
41 bp
rpl2
R6
B
ORF318
R4
R3
R2
R2
atp6
ORF324
R1
R2
R3
R4
R5

Figure S5

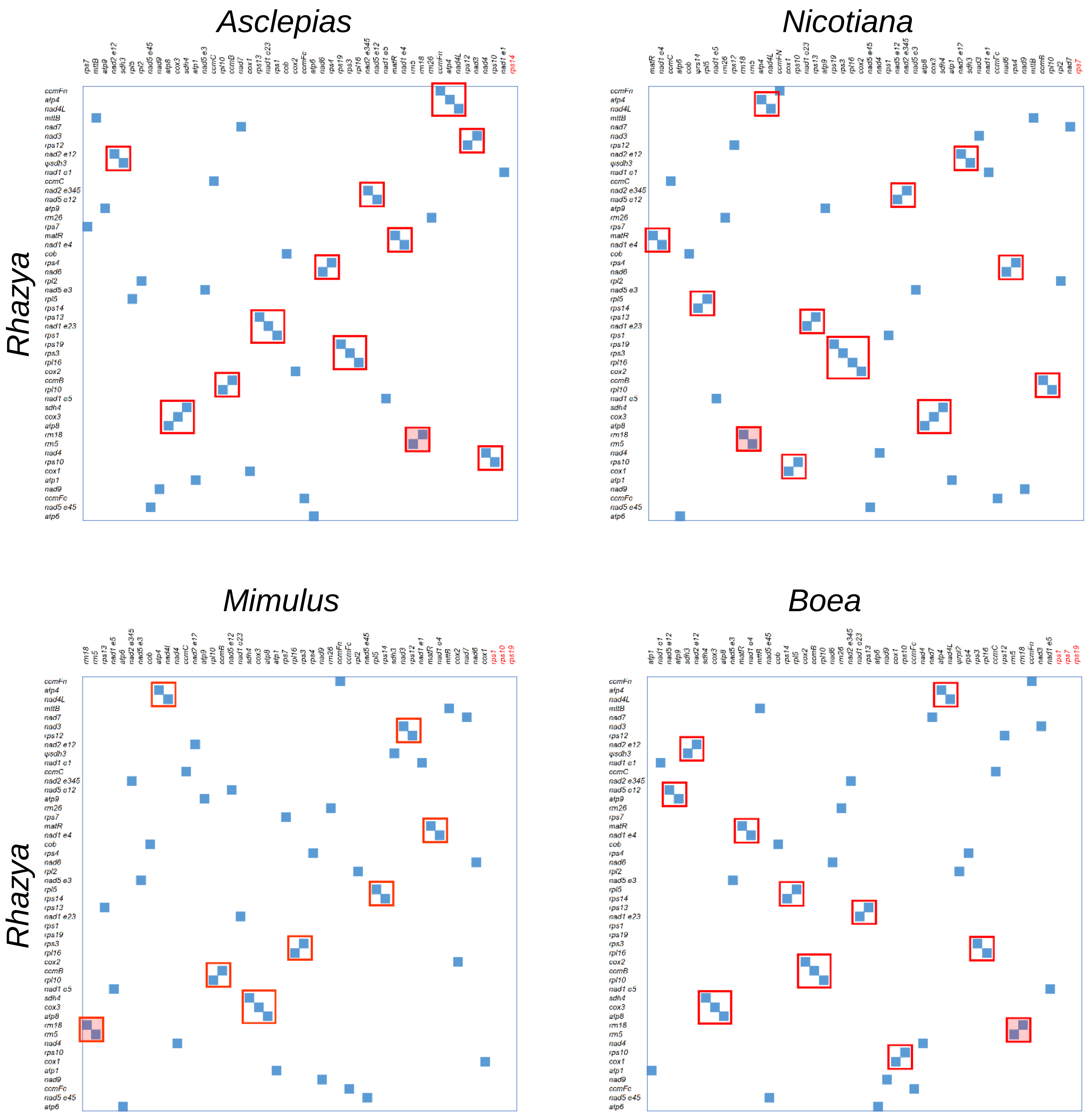

Figure S5

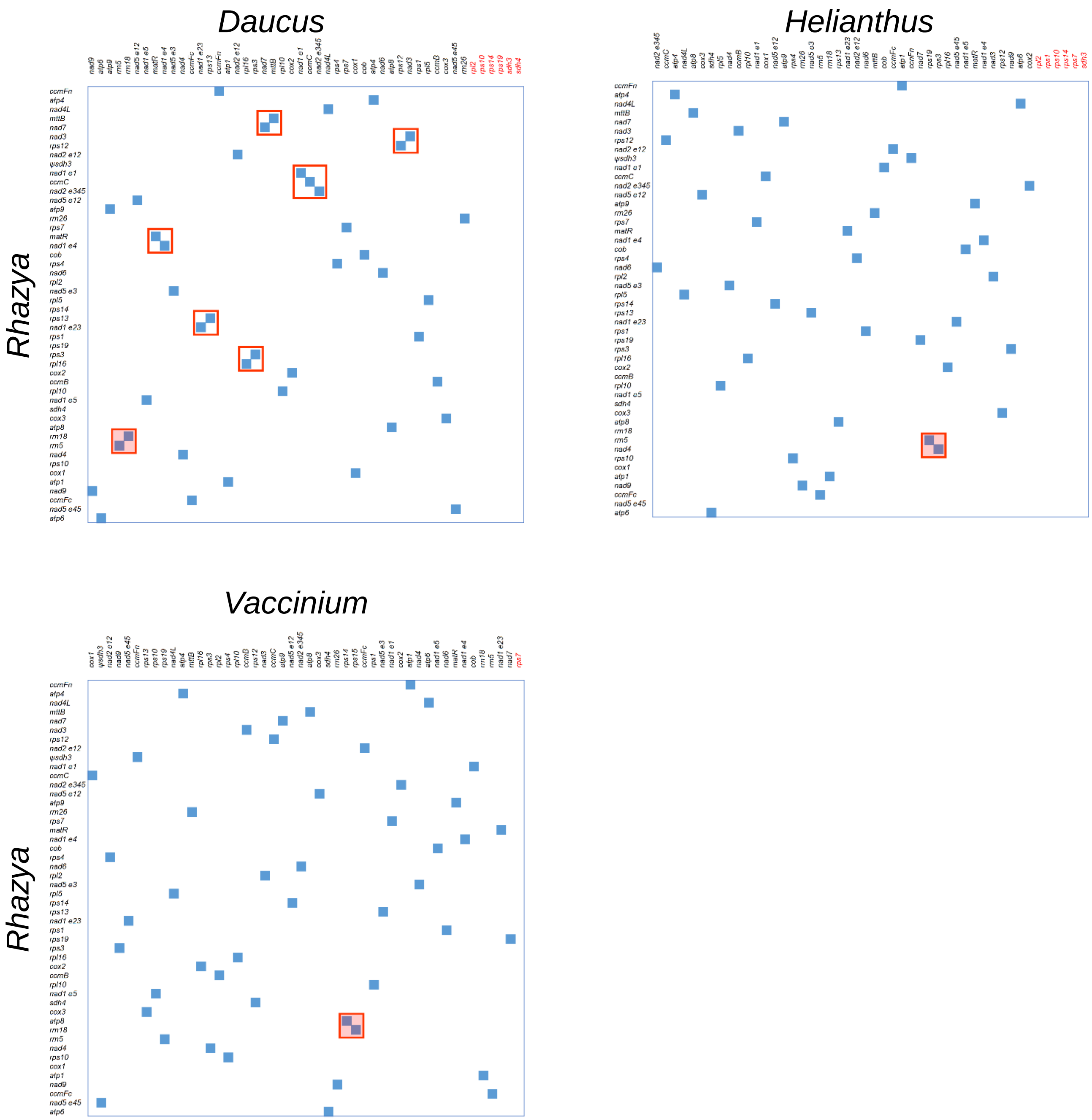
Daucus
Helianthus
Vaccinium
Rhazya
Rhazya
ccmFn
atp4
nad4L
mttB
nad7
nad3
rps12
nad2 e12
ψsdh3
nad1 e1
ccmC
nad2 e345
nad5 e12
atp9
rrn26
rps7
matR
nad1 e4
cob
rps4
nad6
rpl2
nad5 e3
rpl5
rps14
rps13
nad1 e23
rps1
rps19
rps3
rpl16
cox2
ccmB
rpl10
nad1 e5
sdh4
cox3
atp8
rrn18
rrn5
nad4
rps10
cox1
atp1
nad9
ccmFc
nad5 e45
atp6
